# Balancing Positive and Negative Selection: *In Vivo* Evolution of Candida lusitaniae *MRR1*

**DOI:** 10.1128/mBio.03328-20

**Published:** 2021-03-30

**Authors:** Elora G. Demers, Jason E. Stajich, Alix Ashare, Patricia Occhipinti, Deborah A. Hogan

**Affiliations:** a Department of Microbiology and Immunology, Geisel School of Medicine at Dartmouth, Hanover, New Hampshire, USA; b Department of Microbiology & Plant Pathology and Institute for Integrative Genome Biology, University of California—Riverside, Riverside, California, USA; c Dartmouth-Hitchcock Medical Center, Section of Pulmonary and Critical Care Medicine, Lebanon, New Hampshire, USA; University of British Columbia

**Keywords:** *Candida lusitaniae*, Mrr1, evolution, drug resistance, fluconazole, yeast, hydrogen peroxide, chronic infection, cystic fibrosis, *Candida albicans*, *Candida auris*

## Abstract

The evolution of pathogens in response to selective pressures present during chronic infections can influence their persistence and virulence and the outcomes of antimicrobial therapy. Because subpopulations within an infection can be spatially separated and the host environment can fluctuate, an appreciation of the pathways under selection may be most easily revealed through the analysis of numerous isolates from single infections. Here, we continued our analysis of a set of clonally derived *Clavispora* (*Candida*) *lusitaniae* isolates from a single chronic lung infection with a striking enrichment in the number of alleles of *MRR1*. Genetic and genomic analyses found evidence for repeated acquisition of gain-of-function mutations that conferred constitutive Mrr1 activity. In the same population, there were multiple alleles with both gain-of-function mutations and secondary suppressor mutations that either attenuated or abolished the constitutive activity, suggesting the presence of counteracting selective pressures. Our studies demonstrated trade-offs between high Mrr1 activity, which confers resistance to the antifungal fluconazole, host factors, and bacterial products through its regulation of *MDR1*, and resistance to hydrogen peroxide, a reactive oxygen species produced in the neutrophilic environment associated with this infection. This inverse correlation between high Mrr1 activity and hydrogen peroxide resistance was observed in multiple *Candida* species and in serially collected populations from this individual over 3 years. These data lead us to propose that dynamic or variable selective pressures can be reflected in population genomics and that these dynamics can complicate the drug resistance profile of the population.

## Abstract (precis)

Understanding microbial evolution within patients is critical for managing chronic infections and understanding host-pathogen interactions. Here, our analysis of multiple *MRR1* alleles in isolates from a single *Clavispora* (*Candida*) *lusitaniae* infection revealed the selection for both high and low Mrr1 activity.

## INTRODUCTION

Understanding the positive and negative selective pressures that shape drug resistance profiles in microbial populations is critical for combating the development of antimicrobial resistance, an ever-increasing problem in clinical settings. Increased drug resistance in bacteria and fungi has been associated with clinically and agriculturally used antimicrobial agents (reviewed in references 1–3); however, drug resistance elements may also be selected for based on their ability to protect against factors produced by other microbes or plant, animal, and insect hosts (4, 5). Based on the analysis of bacterial isolates, such as Burkholderia dolosa or Pseudomonas aeruginosa, from single patients and across cohorts of patients, it is clear that *in vivo* factors can lead to the repeated selection for subpopulations with the same genes or pathways mutated (6–8). Similar studies have further shown that individual pathways can be upregulated and then downregulated in the same phylogenetic lineages. For example, suppressor mutations within P. aeruginosa *algU* frequently arise in strains already harboring mutations in the gene encoding the AlgU repressor MucA, which causes high AlgU signaling (9). Less is known about the negative selective pressures acting against sustained microbial resistance.

In the study by Demers et al. (10), we described a set of 20 recently diverged *Clavispora* (*Candida*) *lusitaniae* isolates obtained from the lung infection of a single individual with cystic fibrosis (CF). C. lusitaniae is among the emerging non-*albicans Candida* spp. that cause life-threatening disseminated infections in immunocompromised (11–13) and immunocompetent (14, 15) individuals. C. lusitaniae is notorious for its rapid development of resistance to antifungal drugs, including amphotericin B, azoles, and echinocandins (13, 16–19), which is interesting in light of its close phylogenetic relationship to Candida auris (20), a species in which multidrug-resistant isolates have caused hospital-associated outbreaks (21, 22). Our previous analyses of heterogeneity in fluconazole (FLZ) resistance among these isolates identified numerous distinct alleles of *MRR1* (*CLUG_00542*) (10). Multiple alleles encoded gain-of-function (GOF) mutations causing constitutive Mrr1 activity, which, as in other *Candida* species, increased expression of *MDR1* and Mdr1-dependent multidrug efflux pump activity (10, 23–30). At the time that these isolates were recovered, the patient had no history of antifungal treatment, suggesting that selection for constitutively active Mrr1 variants may have been driven by the need for resistance to other host- or microbe-produced compounds. Within this study, however, we found multiple lineages with recently evolved *MRR1* alleles that rendered cells more sensitive to FLZ than even *mrr1*Δ strains. Here, we address the perplexing question of why this population had recently diverged *MRR1* alleles that encoded both high and low Mrr1 activity. To do so, we expressed both native and synthesized *MRR1* alleles that represent intermediates during *MRR1* evolution in a common genetic background and tested the effects of these alleles on growth under *in vivo* relevant conditions. We concluded that multiple C. lusitaniae *MRR1* alleles conferring low Mrr1 activity resulted from an initial mutation that caused constitutive Mrr1 activity followed by a second mutation that either suppressed constitutive activation or inactivated the protein. Constitutive Mrr1 activity caused increased sensitivity to a variety of biologically relevant compounds, including hydrogen peroxide (H_2_O_2_), and suppression of constitutive Mrr1 activity rescued growth under some of these conditions. Monitoring populations from this subject’s respiratory samples over time supports the model that there were opposing selective pressures *in vivo* that selected for and against constitutive Mrr1 activity, as reflected by the trade-off between FLZ and H_2_O_2_ resistance. These data provide insight into the persistence of a heterogeneous fungal population and underscores the complexity and parallelism of evolution that is possible in the human lung during a chronic infection.

## RESULTS

### Naturally evolved C. lusitaniae *MRR1* alleles confer altered Mrr1 activity and FLZ resistance.

**FIG 1:**
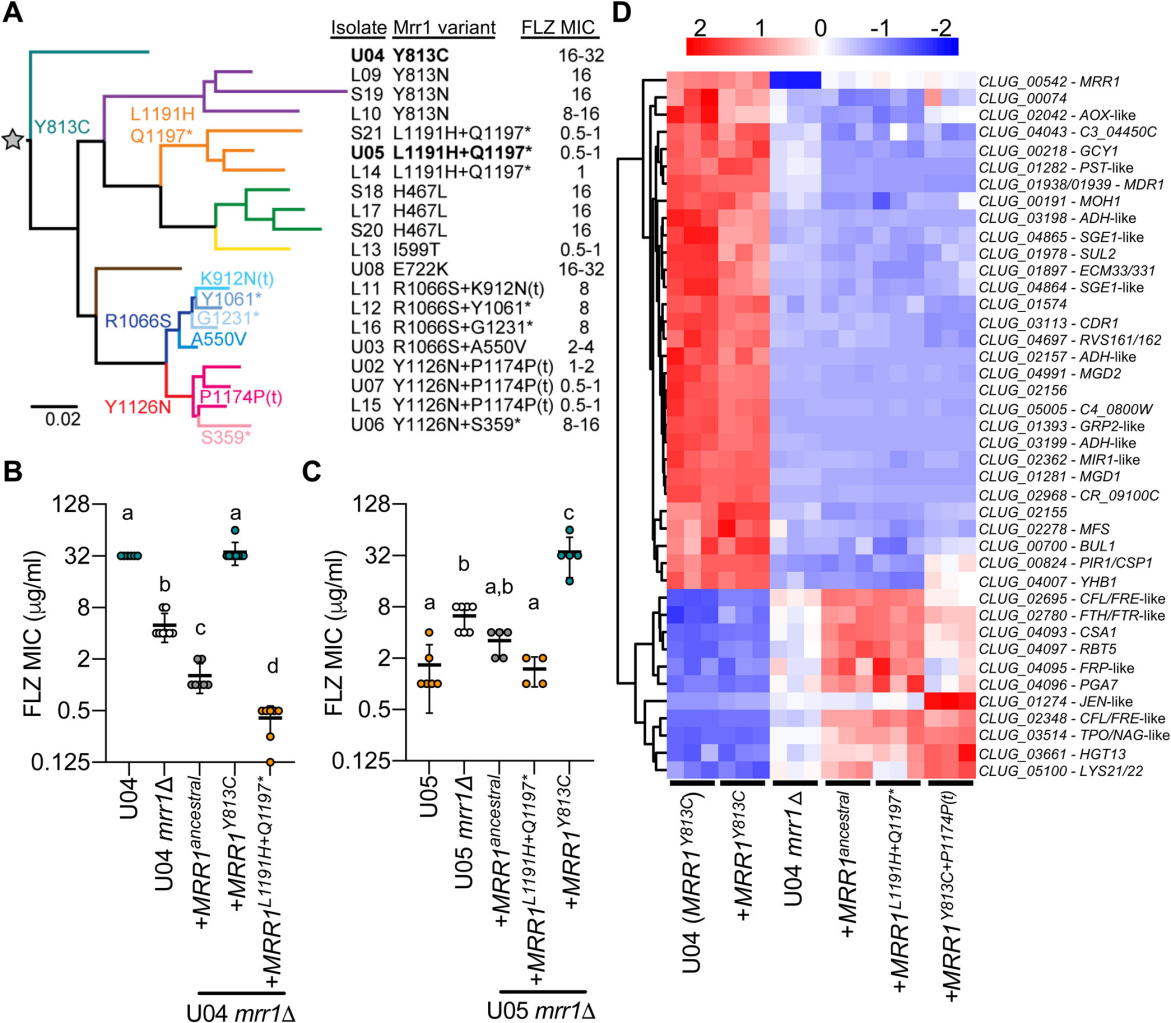
Constitutively active and low-activity Mrr1 variants naturally evolved in a single C. lusitaniae population. (A) Maximum likelihood-based phylogeny constructed from SNPs identified in the whole-genome sequences of 20 C. lusitaniae isolates; modified from Demers et al. (10). Select branchpoints are marked with the Mrr1 variants (text colored to match branches) present in subsequent isolates. Mrr1 variants are identified by amino acid changes that resulted from SNPs or indels; * indicates a stop codon. The one-nucleotide indel in codons P1174 (insertion) and K912 (deletion) cause frameshift mutations that resulted in early termination, denoted with “(t),” at N1176 and L927, respectively. Gray star at the root of the tree denotes the “ancestral” *MRR1* sequence, which lacks any of the mutations listed. U04 and U05, which are used in panels B and C, are highlighted. FLZ MICs (μg/ml) as determined in reference 10 are listed. (B) FLZ MICs for unaltered, *mrr1*Δ and *MRR1* complemented strains in the FLZ-resistant U04 (native allele *MRR1^Y813C^*) strain background. (C) Same as in panel B, but in the FLZ-sensitive U05 strain background (native allele MRR1^L1191H+Q1197^*). Strains containing the same *MRR1* allele in panels B and C are represented by circles of the same color. Data shown represent at least four independent assays on different days. Each sample was statistically compared to every other sample; the same lowercase letters indicate samples that are not significantly different, and different lowercase letters indicate significant differences (*P* < 0.0001 [B] or *P* < 0.001 [C]) as determined by one-way ANOVA with Tukey’s multiple-comparison test of log_2_-transformed values. (D) Differentially expressed genes between strains harboring the constitutively active Mrr1-Y813C variant (U04 and U04 *mrr1*Δ*+MRR1^Y813C^*) and those lacking *MRR1* (U04 *mrr1*Δ) or harboring low-activity variants (U04 *mrr1*Δ*+MRR1^ancestral^* and U04 *mrr1*Δ+MRR1^L1191H+Q1197^*) when grown in liquid YPD medium; statistical cutoffs used were FDR of <0.05 and fold change of ≥2 (see Table S1 in the supplemental material). Normalized counts per million (CPM) from RNA-Seq are scaled by row (gene) with hierarchical clustering by Euclidean distance. Complemented strains are denoted by their respective *MRR1* allele. Predicted C. albicans homologs are listed next to C. lusitaniae gene names (Table S1).

Each of the 20 closely related C. lusitaniae isolates from a single individual contained at least one nonsynonymous single nucleotide polymorphism (SNP) or single nucleotide insertion or deletion (indel) in *MRR1* relative to the deduced *MRR1* sequence of their most recent common ancestor (*MRR1^ancestral^*), the sequence that resulted upon removing any of the SNPs that varied across the population (Fig. 1A) (10). While we did not identify any isolates with the *MRR1^ancestral^* sequence in samples from this subject, we did find an isolate from another patient (B_L06) that contained the same sequence as the “*MRR1^ancestral^*” allele. To determine the impact of specific mutations in *MRR1* on Mrr1 activity, we expressed different *MRR1* alleles in a common genetic background in which the native *MRR1* had been deleted (U04 *mrr1*Δ). Deletion of *MRR1* in the FLZ-resistant strain U04 reduced the FLZ MIC from 32 μg/ml to 4 μg/ml (10), and the decrease in MIC was complemented by restoring the native *MRR1^Y813C^* allele (Fig. 1B). We previously published that FLZ resistance in isolates from Fig. 1A correlated with expression of *MDR1* (10), also known as *MFS7* (19), and here show that deletion of *MDR1* similarly reduced the FLZ MIC of unaltered U04 and U04 *mrr1*Δ +*MRR1^Y813C^* (8- to 16-fold) (see Fig. S1A in the supplemental material). Complementation of U04 *mrr1*Δ with the *MRR1^ancestral^* allele resulted in a FLZ MIC of 1 μg/ml, 4-fold lower than the FLZ MIC of U04 *mrr1*Δ, suggesting that Mrr1^ancestral^ had a function that reduced the FLZ MIC (Fig. 1B). Expression of an *MRR1* allele from a closely related FLZ-sensitive isolate (MRR1^L1191H+Q1197^*) reduced the FLZ MIC to 0.5 μg/ml (Fig. 1B). Similar relationships between the *MRR1* allele and FLZ MIC were observed when the *MRR1^ancestral^*, *MRR1^Y813C^*, and MRR1^L1191H+Q1197^* alleles were expressed in a *mrr1*Δ derivative of the FLZ-sensitive strain U05 (Fig. 1C); thus, further analyses were performed in the U04 background alone.

10.1128/mBio.03328-20.1 FIG S1 Deletion of *MRR1* causes an Mdr1-dependent increase in FLZ resistance. (A) FLZ MIC of U04-derived strains containing *MRR1* (teal shapes), lacking *MRR1* (open shapes), and/or lacking *MDR1* (squares). Data shown represent at least three independent assays on different days. Each sample was statistically compared to every other sample; the same lowercase letters indicate samples that are not significantly different, and different lowercase letters indicate significant differences (b and c, *P* < 0.05; all other pairwise comparisons, *P* < 0.0001) as determined by one-way ANOVA with Tukey’s multiple-comparison test of log_2_-transformed values. (B) FLZ MICs for unaltered wild type (WT, closed circles) and *mrr1*Δ (open circles) mutants. U04 (FLZ-resistant; teal) and U05 (FLZ-sensitive; orange) are closely related to each other but distantly related to the FLZ-sensitive ATCC 42720 and DH2383 strains. Data shown represent three independent assays on different days. Two-way ANOVA with Sidak’s multiple-comparison test of log_2_-transformed values between WT and *mrr1*Δ for each strain; ***, *P* < 0.001. (C) FLZ MICs of unaltered U05, U05 *mrr1*Δ (*MRR1* replaced by the selectable marker *NAT1*), and two independent clones of U05 with *NAT1* expressed from an intergenic site on chromosome 4. Data shown represent three independent assays on different days. Each sample was statistically compared to every other sample; the same lowercase letters indicate samples that are not significantly different, and different lowercase letters indicate significant differences (*P* < 0.01) as determined by one-way ANOVA with Tukey’s multiple-comparison test of log_2_-transformed values. Download FIG S1, TIF file, 0.3 MB. Copyright © 2021 Demers et al. 2021 Demers et al. https://creativecommons.org/licenses/by/4.0/ This content is distributed under the terms of the Creative Commons Attribution 4.0 International license.

10.1128/mBio.03328-20.7 TABLE S1 Genes differentially regulated by constitutively active Mrr1. Sheet A shows the subset of genes differentially regulated by constitutively active Mrr1; genes with a fold change of >2 (FDR < 0.05) are highlighted in dark red (upregulated) or blue (downregulated) and genes with a fold change between 1.5 to 2 (FDR < 0.05) are similarly highlighted in lighter colors. Sheet B shows the complete linear model results with normalized CPMs for each strain and gene. Download Table S1, XLSX file, 2.4 MB. Copyright © 2021 Demers et al. 2021 Demers et al. https://creativecommons.org/licenses/by/4.0/ This content is distributed under the terms of the Creative Commons Attribution 4.0 International license.

RNA-sequencing (RNA-seq) analysis validated the previously published result that *MDR1* expression paralleled FLZ MIC (Fig. 1D and Table S1) (10). Comparison of gene expression profiles for U04 (*MRR1^Y813C^*), U04 *mrr1*Δ, and U04 *mrr1*Δ+*MRR1^Y813C^* found that *mrr1*Δ was fully complemented upon return of *MRR1^Y813C^* to the native locus (Fig. 1D and S2A) and confirmed that Mrr1 appears to both positively and negatively regulate a subset of genes (10, 29). Furthermore, a correlation analysis found that gene expression in U04 *mrr1*Δ+*MRR1^ancestral^* and U04 *mrr1*Δ+MRR1^L1191H+Q1197^* was similar but distinct from that of the *mrr1*Δ strain (Fig. 1D and S2A). A linear model comparing these strains identified 41 genes with at least a 2-fold change in expression and corrected *P* value of <0.05. Comparison of nonisogenic C. lusitaniae strains similarly identified 22 of the genes in Table S1 as putatively Mrr1 regulated, including *MRR1* itself (10, 29). Eighteen genes were homologs or had similar predicted functions as genes previously published as regulated by Candida albicans Mrr1 (24), including *MDR1*, *FLU1*, and multiple putative methylglyoxal reductases encoded by *GRP2*-like genes, such as *MGD1* and *MGD2* (Fig. 1D and Table S1). Other genes within the Mrr1 regulon are discussed further below. These data indicate that the Mrr1-ancestral and Mrr1-L1191H+Q1197* variants had low basal activity, while Mrr1-Y813C was constitutively active.

10.1128/mBio.03328-20.2 FIG S2 C. lusitaniae Mrr1 acts as a positive and negative regulator of gene expression. (A) Pairwise comparisons of normalized CPM for U04, U04 *mrr1*Δ, or U04 *mrr1*Δ complemented strains denoted by their respective *MRR1* allele; datapoints colored to match strains on the *y* axis. *R*^2^ from linear regression shown for each comparison. (B) Subset of Mrr1-regulated genes from Fig. 1D with at least 2-fold higher expression in U04 *mrr1*Δ than U04 *mrr1*Δ+ *MRR1^ancestral^*; complemented strains are denoted as by their respective *MRR1* alleles. Download FIG S2, TIF file, 0.4 MB. Copyright © 2021 Demers et al. 2021 Demers et al. https://creativecommons.org/licenses/by/4.0/ This content is distributed under the terms of the Creative Commons Attribution 4.0 International license.

The unexpected finding that FLZ MIC was higher upon deletion of *MRR1* than U04 expressing *MRR1^ancestral^* or U05 was also observed in distantly related C. lusitaniae strains, ATCC 42720 and DH2383, with FLZ MICs of ∼1 to 2 μg/ml (Fig. 1B and Fig. S1B). In both cases, deletion of *MRR1* led to a 2- to 4-fold increase in FLZ MIC (Fig. S1B). The elevated FLZ MIC in *mrr1*Δ strains was *MDR1* dependent, as the FLZ MIC was even lower in U04 *mrr1*Δ *mdr1*Δ than in U04 *mrr1*Δ (Fig. S1A). The increase was not due to introduction of the selectable marker *NAT1*, which encodes a nourseothricin acetyltransferase (31), as expression of *NAT1* from an intergenic site in the FLZ-sensitive U05 strain did not alter the FLZ MIC (Fig. S1C). These data led us to hypothesize that some Mrr1 variants (Mrr1-Y813C) caused constitutively high *MDR1* expression, while other Mrr1 variants (both Mrr1-ancestral and the recently diverged Mrr1-L1191H+Q1197*) repressed the expression of at least some Mrr1-controlled genes, such as *MDR1*. Indeed, the RNA-Seq analysis identified six genes, including *MDR1*, that while positively regulated when Mrr1 was constitutively active, were more highly expressed in U04 *mrr1*Δ than in those strains encoding low-activity Mrr1 variants (Fig. 1D and S2B). These data suggest that, for a small subset of Mrr1-regulated genes, including *MDR1*, low-activity Mrr1 variants may directly or indirectly inhibit expression. Cap1 (Clug_02670), another transcription factor known to regulate *MDR1* in C. albicans (32, 33), was not responsible for the increase in *MDR1* expression in the absence of *MRR1*, as deletion of *CAP1* from U05 *mrr1*Δ did not alter the FLZ MIC of this strain (4 to 8 μg/ml, *n* = 3).

### Premature termination had varied effects on Mrr1 activity and inducibility in clinical isolates.

**FIG 2:**
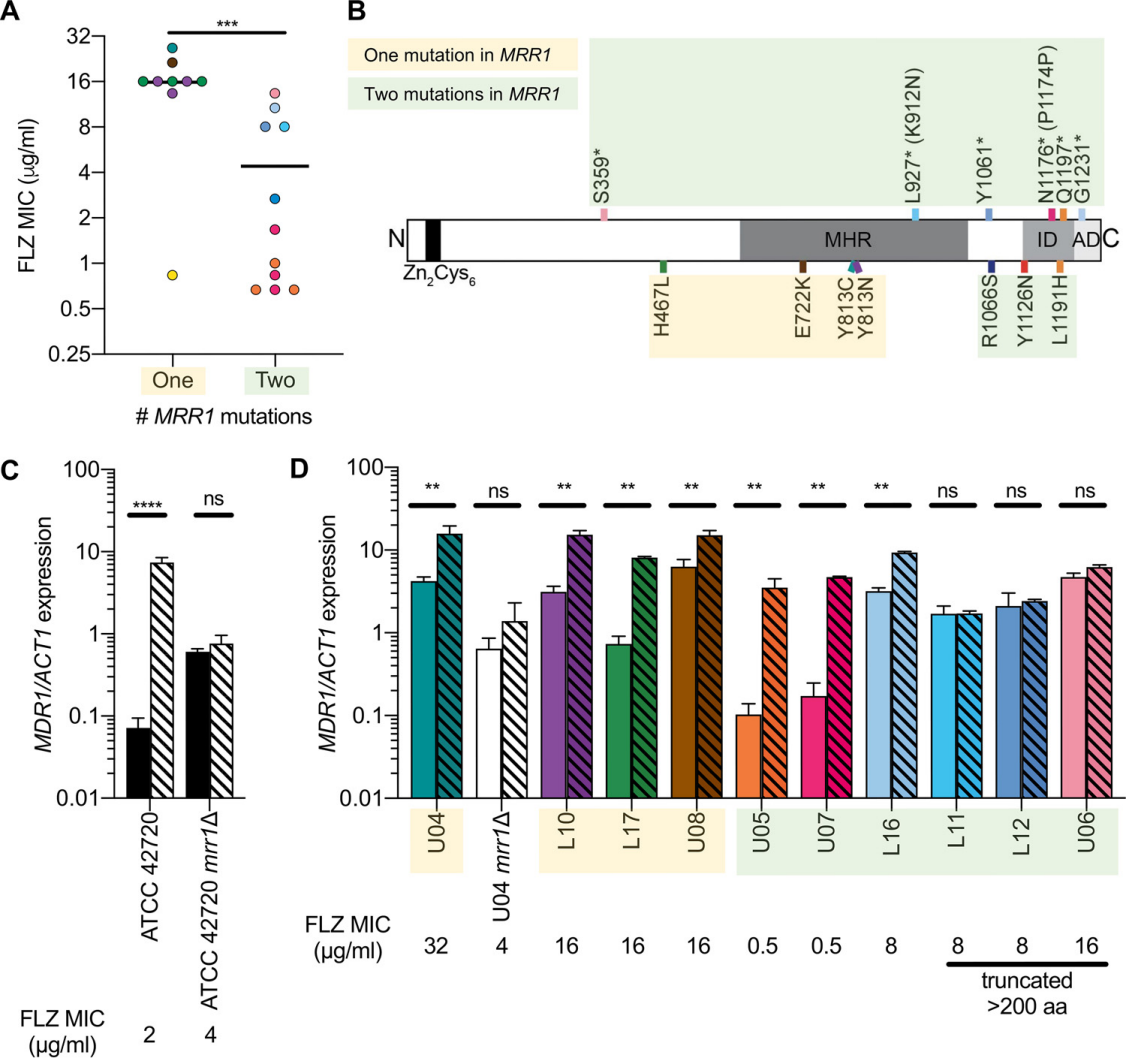
Premature stop codons in Mrr1 differentially impact *MDR1* induction by benomyl. (A) Mean FLZ MICs for each of the 20 clinical C. lusitaniae isolates in Fig. 1A separated by the number of nonsynonymous mutations within *MRR1* (as defined in reference 10); mean of each group is shown. Two-tailed unpaired *t* test of log_2_-transformed MIC values; ***, *P* < 0.001. (B) Schematic of C. lusitaniae *MRR1* annotated with putative regulatory domains determined by sequence analysis and homology to C. albicans (22) and locations of truncating (top) and putative activating (bottom) mutations. Putative domains include a DNA binding domain with a zinc cluster motif (Zn_2_Cys_6_; amino acids 33 to 61), a transcriptional regulatory middle homology region (MHR; amino acids ∼607 to 1023), an inhibitory domain (ID; amino acids 1123 to 1217), and an activating domain (AD; amino acids 1218 to 1265). L927 and N1176 are the locations of stop codons caused by indels in codons K912 and P1174, respectively. (C and D) *MDR1* expression normalized to *ACT1* in the absence (solid bars) or presence (striped bars) of 50 μg/ml benomyl. Means ± standard deviations (SDs) of representative data in biological triplicates are shown; similar trends observed on at least three different days. Two-way ANOVA with Sidak’s multiple-comparison test; **, *P* < 0.01; ****, *P* < 0.0001; ns, not significant. In panel D, strain names are highlighted corresponding to the number of mutations in *MRR1*, yellow for one and green for two, as in Fig. 2B. The colors of the circles (A), lines (B), and bars (D) correspond to *MRR1* alleles shown Fig. 1A.

Each of the 20 sequenced C. lusitaniae isolates contained *MRR1* alleles with either one or two nonsynonymous mutations relative to *MRR1^ancestral^* (Fig. 1A), and we found that C. lusitaniae isolates with two mutations in *MRR1* had a significantly lower average FLZ MIC than isolates with a single *MRR1* mutation (Fig. 2A). Interestingly, six of the seven *MRR1* alleles in the “two-mutation” set had premature stop codons, resulting in the loss of 34 to 906 amino acids (Fig. 2B). There were two instances in which the same mutation was found in combination with different nonsense mutations (*) or single nucleotide indels that led to early termination (t): *MRR1^**Y1126N**+P1174P(t)^* or MRR1^**Y1126N**+S359^* and *MRR1^**R1066S**^*^+^*^K912N(t)^*, MRR1^**R1066S**+Y1061^*, or MRR1^**R1066S**+G1231^* (common mutations in bold) (Fig. 1A). This suggested a complex evolutionary history for these alleles.

To better understand the effects of mutations in *MRR1* on Mrr1 activity, we analyzed the effects of a chemical inducer of Mrr1 activity, benomyl (24, 34, 35), on *MDR1* expression. Benomyl strongly induced *MDR1* expression in an Mrr1-dependent manner in the FLZ-sensitive strain ATCC 42720 (Fig. 2C) and, to a lesser extent, in the FLZ-resistant strain U04, which had high basal *MDR1* expression (Fig. 2D). Quantitative reverse transcription-PCR (qRT-PCR) analysis of *MDR1* expression and induction by benomyl in this collection of clinical isolates with different Mrr1 variants found that the two isolates with the lowest basal *MDR1* expression and lowest FLZ MIC (U05 and U07) had the greatest induction by benomyl (34- and 27-fold, respectively) (Fig. 2D), suggesting that despite the loss of 68 or 89 amino acids, respectively, they encoded functional but low-activity Mrr1 variants. Three isolates, L11, L12, and U06, had intermediate FLZ MICs and *MDR1* expression levels but did not show benomyl induction, similar to *mrr1*Δ strains (Fig. 2C and D). These strains all encoded Mrr1 variants lacking greater than 200 amino acids, suggesting that these mutations caused a loss of Mrr1 function. Isolate L16, which encoded two mutations in Mrr1 but only lacked 34 amino acids from the C terminus, phenocopied strains with a single mutation in *MRR1*, such as U04, L10, L17, and U08, suggesting either removal of a C-terminal regulatory region or that the mutation not causing premature termination increased Mrr1 activity (Fig. 2D).

### Premature stop codons repeatedly arose in constitutively active Mrr1 variants and caused either a loss of constitutive Mrr1 activity or a complete loss of Mrr1 function.

**FIG 3:**
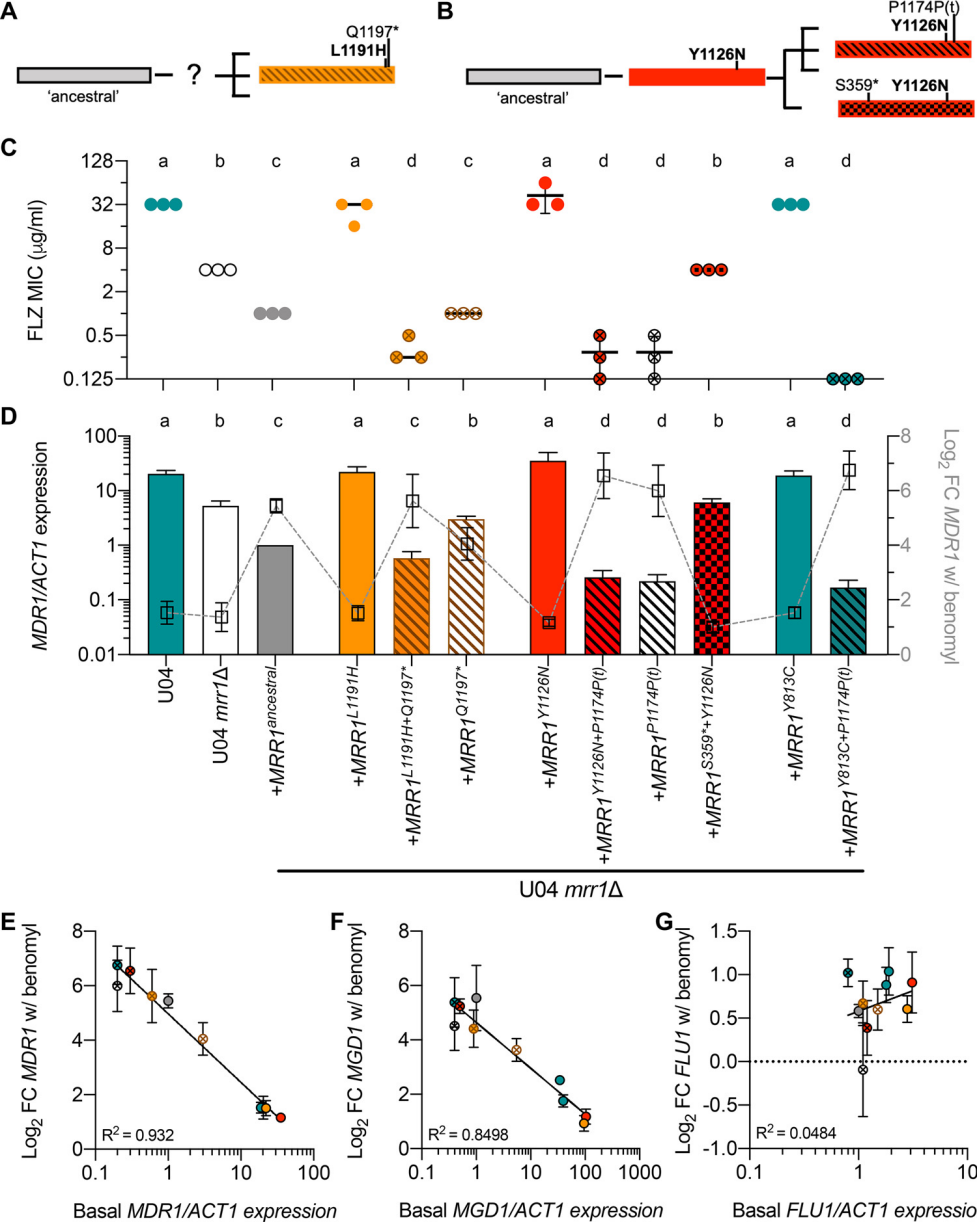
Premature stop codons repeatedly arose in constitutively active Mrr1 variants resulting in reduced Mrr1 activity but, in some cases, restored Mrr1 inducibility. Schematic of inferred evolution of *MRR1* alleles in the L1191H+Q1197* (A) and Y1126N (B) lineages. (C) FLZ MICs for U04, U04 *mrr1*Δ and *MRR1* complemented strains in the U04 *mrr1*Δ background. Means ± SDs from three independent assays on different days shown. Each sample was statistically compared to every other sample; the same lowercase letters indicate samples that are not significantly different, and different lowercase letters indicate significant differences (*P* < 0.01) as determined by one-way ANOVA with Tukey’s multiple-comparison test of log_2_-transformed values. (D) *MDR1* expression normalized to *ACT1* from culture grown in YPD (bars, left *y* axis). Means ± SDs from three independent assays on different days; data from each day were normalized to the expression of U04 *mrr1*Δ+*MRR1^ancestral^*. Each sample was statistically compared to every other sample; the same lowercase letters indicate samples that are not significantly different, and different lowercase letters indicate significant differences (b to d, *P* < 0.05; all other pairwise comparisons, *P* < 0.01) as determined by one-way ANOVA with Tukey’s multiple-comparison testing of log_2_-transformed data. Overlaid with log_2_-transformed mean ± SD fold change (FC) in normalized *MDR1* expression following exposure to 50 μg/ml benomyl (squares, right *y* axis); full data presented with statistics in Fig. S3D. (C and D) FLZ MICs and *MDR1/ACT1* expression data are colored to match; the sample names are shown on the *x* axis of panel D. Comparison of mean basal *MDR1* (E), *MGD1* (F), or *FLU1* (G) expression from panel D and Fig. S3B or C, respectively, excluding strains lacking functional *MRR1*, and mean ± SD log_2_-transformed FC of the induction following benomyl exposure from Fig. S3D to F; circles colored to match those in panel C. Goodness of fit *r*^2^ value for nonlinear regression shown.

In light of the mixed effects that these two-mutation *MRR1* alleles had on Mrr1 activity, we sought to determine the individual effects of mutations within each allele with a focus on the two strains with the lowest basal *MDR1* expression and the strongest induction of *MDR1* in response to benomyl, MRR1^L1191H+Q1197^* (in U05) (Fig. 3A) and *MRR1^Y1126N+P1174P(t)^* (in U07) (Fig. 3B). We found that the *MRR1^L1191H^* mutation caused a 32-fold increase in FLZ MIC (Fig. 3C) and 22-fold increase in *MDR1* expression (Fig. 3D) compared to that for *MRR1^ancestral^*, indicating that, like the Mrr1-Y813C variant, Mrr1-L1191H was constitutively active. In contrast, MRR1^Q1197^*, which caused the loss of 68 amino acids from the C terminus of Mrr1, did not significantly alter the FLZ MIC compared to that for the *MRR1^ancestral^* allele, suggesting that it was neither a constitutively activating nor null mutation (Fig. 3C). The combination of mutations in MRR1^L1191H+Q1197^* resulted in a 128-fold decrease in FLZ MIC (Fig. 3C) and 38-fold decrease in *MDR1* expression (Fig. 3D) compared to that for a strain expressing *MRR1^L1191H^*, phenotypes that mirrored the strain expressing *MRR1^ancestral^*.

10.1128/mBio.03328-20.3 FIG S3 The C terminus of Mrr1 is required for constitutive expression, but not induction by benomyl, of multiple Mrr1-regulated genes. (A) Schematic of C. lusitaniae Mrr1 activation. In the absence of an inducer, Mrr1 variants, such as the Mrr1-ancestral variant, are in a low-activity or uninduced state (top left). The presence of an inducer (top right), such as benomyl, or mutations (red star) that cause constitutive Mrr1 activity (bottom left), increase the activity of Mrr1 and expression of Mrr1-regulated genes. Removal of <100 amino acids from the C terminus of constitutively active Mrr1 variants returns them to a low-activity but still inducible state (bottom right). Log_2_-transformed expression of *MGD1* (*CLUG_01281*) (B) or *FLU1* (*CLUG_05825*) (C) normalized to *ACT1*. Log_2_ fold change (FC) in *MDR1* (D), *MGD1* (E) or *FLU1* (F) expression, normalized to *ACT1*, with 50 μg/ml benomyl compared to the no-benomyl controls shown in Fig. 3D and panels B and C, respectively. For panels B to F, the same lowercase letters indicate samples that are not significantly different, and different lowercase letters indicate significant differences (a and b, *P* < 0.05), as determined by one-way ANOVA with Tukey’s multiple-comparison testing between strains encoding the Mrr1-ancestral variant, the three constitutively active variants, and the five prematurely terminated variants. Data shown represent three independent assays on different days; data from each day were normalized to the expression of U04 *mrr1*Δ+*MRR1^ancestral^* and are connected by lines. All samples are color coded to match across all graphs and those in Fig. 3C and D; the sample names are shown on the *x* axes in panels C and F. Download FIG S3, TIF file, 1.0 MB. Copyright © 2021 Demers et al. 2021 Demers et al. https://creativecommons.org/licenses/by/4.0/ This content is distributed under the terms of the Creative Commons Attribution 4.0 International license.

*MRR1^Y1126N+P1174P(t)^* (from U07) and MRR1^Y1126N+S359^* (from the closely related U06) (Fig. 1A), were similarly analyzed (Fig. 3B). Expression of *MRR1^Y1126N^* in U04 *mrr1*Δ caused high FLZ resistance (MIC of 32 to 64 μg/ml) (Fig. 3C) and *MDR1* expression (Fig. 3D), similar to that for strains with *MRR1^Y813C^* or *MRR1^L1191H^*. Addition of the frameshift-inducing indel at P1174, which causes a premature stop codon at N1176 removing 89 amino acids, yielding *MRR1^Y1126N+P1174P(t)^*, caused a 128-fold decrease in FLZ MIC and >100-fold decrease in *MDR1* expression relative to that for the strain expressing *MRR1^Y1126N^*, again leading to a strain that phenocopied one expressing *MRR1^ancestral^* (Fig. 3C and D). The addition of the indel at P1174 to an allele with a different constitutively active variant, creating *MRR1^Y813C+P1174P(t)^*, similarly caused 256- and >100-fold decreases in FLZ MIC and *MDR1* expression, respectively, relative to that for a strain expressing the *MRR1^Y813C^* allele (Fig. 3C and D). Further RNA-Seq analysis of U04 *mrr1*Δ+*MRR1^Y813C+P1174P(t)^* showed decreased expression of many genes positively regulated by Mrr1 (Fig. 1D). In contrast, addition of a SNP causing an early stop codon at S359 to the allele with the activating Y1126N mutation (MRR1^Y1126N+S359^*) yielded a strain that phenocopied U04 *mrr1*Δ, indicating this variant, lacking 906 amino acids from the C terminus, was inactive (Fig. 3C and D). Together, these data show that the Y1126N mutation, which arose first, caused constitutive Mrr1 activity that was subsequently suppressed by premature stop codons that either restored low activity [P1174P(t)] or eliminated activity (S359*).

In addition to the differences in basal activity, the individual mutations alone and in combination affected chemical inducibility by benomyl. Levels of *MDR1* were strongly induced by benomyl in U04 *mrr1*Δ+*MRR1^ancestral^* (40-fold increase) but not in the U04 parental strain with high Mrr1 activity or its *mrr1*Δ derivative (Fig. 3D). The three constitutively active variants (Mrr1-Y813C, Mrr1-L1191H, and Mrr1-Y1126N) showed only a 2- to 3-fold increase in *MDR1* expression with benomyl (Fig. 3D), similar to what was observed for FLZ-resistant clinical isolates (Fig. 2D). Surprisingly, addition of mutations that caused premature stop codons within the last 100 amino acids of Mrr1 to the constitutively active variants restored inducibility by benomyl (Fig. 3D). In fact, there was a significant inverse correlation between basal *MDR1* expression and fold induction by benomyl (Fig. 3E).

As in C. albicans, C. lusitaniae Mrr1 regulates the expression of the methylglyoxal reductase encoded by *MGD1* (*CLUG_01281* or *GRP2*) (10, 24, 29, 36, 37) and the multidrug efflux pump encoded by *FLU1* (*CLUG_05825*) (10, 29, 38, 39) (Table S1 and Fig. S3A). As with *MDR1*, expression of both *MGD1* and *FLU1* was significantly higher in strains encoding constitutively active variants than in a strain encoding the Mrr1 ancestral variant, and the absence of the C terminus in strains with activating mutations caused a significant decrease in basal expression (Fig. S3B and S3C). Benomyl induction of *MGD1*, like that of *MDR1* (Fig. S3D), was restored upon loss of the C terminus from constitutively active Mrr1 variants, further supporting the strong negative correlation between basal expression and benomyl inducibility (Fig. 2F and Fig. S3E). *FLU1* expression, however, was not induced by benomyl in any strain, suggesting that *FLU1* regulation by Mrr1 differs from *MGD1* and *MDR1* regulation (Fig. 2G and Fig. S3F). Furthermore, *MDR1* and *MGD1* were both derepressed in the absence of Mrr1 (Fig. 1D and Fig. S2B), while *FLU1* was not and was only weakly differentially expressed between strains with constitutive and low Mrr1 activity (<2-fold) (Fig. S3C). Together these data indicate the C terminus of Mrr1 is required for constitutive expression of multiple Mrr1-regulated genes but not for benomyl induction of the Mrr1-regulated genes tested (Fig. S3A). Combined with the Mrr1 activity across clinical isolates (Fig. 2D), these data indicate that in some strains with constitutively active Mrr1 variants, there was repeated selection for mutations to decrease Mrr1 activity, resulting in a mixed population containing constitutively active, prematurely terminated but inducible and loss-of-function (LOF) Mrr1 variants.

### Constitutive Mrr1 activity negatively impacts H_2_O_2_ resistance.

**FIG 4:**
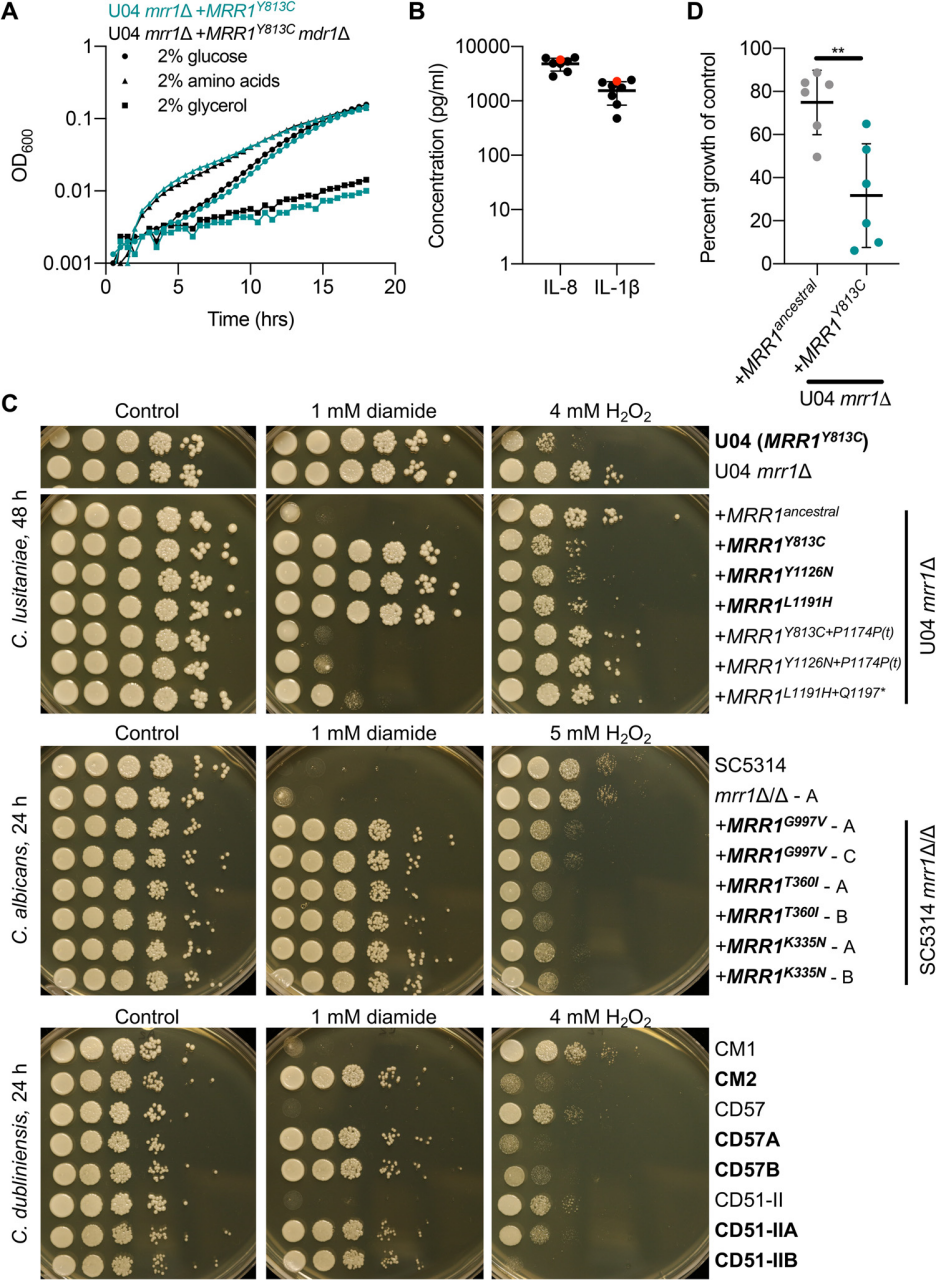
Constitutive Mrr1 activity decreases resistance to H_2_O_2_ in multiple *Candida* species. (A) Growth curve of U04 *mrr1*Δ+*MRR1^Y813C^* (teal) and U04 *mrr1*Δ+*MRR1^Y813C^ mdr1*Δ (black) grown at 37°C in YNB medium supplemented with the indicated carbon source: glucose (circles), amino acids (triangles), or glycerol (squares). Means from representative data acquired in triplicates shown. (B) Quantification of cytokines IL-8 and IL-1β in BAL fluid from the CF patient with (red) or seven patients without (black) C. lusitaniae in their lungs. Two-way ANOVA with Sidak’s multiple-comparison testing found no significant differences. (C) Serial dilution assays of C. lusitaniae, C. albicans, and C. dubliniensis strains on YPD or YPD supplemented with the indicated concentration of diamide or H_2_O_2_. Strain names in bold font were shown to contain GOF mutations in Mrr1 resulting in increased FLZ resistance (Fig. 3C and references 39, 45, and 46). Plates were imaged after 24 or 48 h of growth at 37°C, as indicated. (D) Percent growth in well-aerated 5 ml YPD plus 1 mM H_2_O_2_ was calculated relative to that of the vehicle only control after 22 to 24 h growth at 37°C. These data represent six independent assays performed on different days. Significance determined by paired *t* test; **, *P* < 0.01.

We next sought to understand why mutations that reduce Mrr1 activity might repeatedly arise in this chronic infection. Previous studies have shown that overexpression of drug efflux pumps in drug-resistant microbes can cause a fitness defect due to the energetic cost of constitutive pump production and activity in the absence of a selective substrate (40–42). Deletion of *MDR1* from U04 *mrr1*Δ+*MRR1^Y813C^*, which constitutively expresses *MDR1*, however, did not alter the growth kinetics (Fig. 4A). In the absence of an obvious growth defect, we considered factors present in the CF lung, which has been characterized as a highly inflamed environment containing elevated levels of neutrophils, macrophages, and oxidative stress (reviewed in references 43 and 44). Although little is known about fungus-dominated chronic lung infections in CF, such as the infection from which these isolates were obtained, an analysis of cytokines within the bronchoalveolar lavage (BAL) fluid from the patient these isolates originated from showed proinflammatory cytokines (interleukin 8 [IL-8] and IL-1β) present were consistent with the neutrophilic environment seen in other patients with CF (Fig. 4B) (44).

In light of these findings, we investigated the effects of Mrr1 activity on reactive oxygen species (ROS) stress generated by H_2_O_2_, a stress strongly associated with high neutrophil counts. In a serial dilution assay, we found that isogenic strains harboring constitutively active Mrr1 variants, while resistant to FLZ and diamide (Fig. S4A), had increased sensitivity to 4 mM H_2_O_2_ compared to that of one harboring the Mrr1 ancestral variant (Fig. 4C). Diamide was used to illustrate relative Mrr1 activity instead of FLZ, because serial dilution assays on rich medium (yeast-peptone-glucose [YPD]) containing FLZ are not always representative of FLZ MIC, which are assessed in defined medium (Fig. S4B). Secondary mutations resulting in either a phenotype associated with low Mrr1 activity that is inducible or a complete loss of Mrr1 activity both restored H_2_O_2_ resistance to levels similar to that of strains harboring the Mrr1 ancestral variant (Fig. 4C; Fig. S4A). The effects of Mrr1 activity on H_2_O_2_ sensitivity were similar among isogenic strains in both the U04 and U05 backgrounds (Fig. 4C and Fig. S4B). Surprisingly, deletion of *MDR1* from strains harboring the constitutively active Mrr1-Y813C variant partially rescued growth (Fig. S4A); however, the absence of *MDR1* did not completely explain the differences, as strains lacking *MRR1* had increased H_2_O_2_ resistance despite elevated *MDR1* expression (Fig. 4C). Additionally, the double mutant U04 *mrr1*Δ *mdr1*Δ did not have increased resistance to H_2_O_2_ compared to that of U04 *mrr1*Δ (Fig. S4A) suggesting this may be a complex response. A secondary assay quantifying growth after ∼24 h in liquid cultures containing 1 mM H_2_O_2_, though variable day to day, confirmed there was a reproducible difference in growth between strains harboring the low-activity Mrr1 ancestral and constitutively active Mrr1-Y813C variants (Fig. 4D). To determine if this phenomenon was unique to C. lusitaniae, we examined a set of isogenic C. albicans isolates (39) and *in vivo*- or *in vitro*-evolved Candida dubliniensis isolates (25) expressing *MRR1* alleles containing GOF mutations. We found that for all C. albicans and C. dubliniensis strain sets tested, strains with high Mrr1 activity, which had increased FLZ (39, 45, 46) and diamide resistance, were more sensitive to H_2_O_2_ than strains with low Mrr1 activity or lacking *MRR1* (Fig. 4C). As in C. lusitaniae, deletion of *MDR1* restored growth on H_2_O_2_ in C. albicans (Fig. S4C). These data show that the Mrr1 activity-driven trade-off between FLZ and H_2_O_2_ resistance is conserved across multiple *Candida* species.

10.1128/mBio.03328-20.4 FIG S4 Loss of or decreased Mrr1 activity and deletion of *MDR1* restore H_2_O_2_ resistance in multiple C. lusitaniae strains and *Candida* species. Serial dilution assays of C. lusitaniae strain U04 (FLZ-resistant) (A) and U05 (FLZ-sensitive) (B) with constitutive or low Mrr1 activity on YPD or YPD supplemented with 8 μg/ml FLZ, 1 mM diamide, or the indicated concentration of H_2_O_2_. Similar results seen on at least three separate days. (C) Serial dilution of C. albicans strain SC5314 *mrr1*Δ/Δ expressing the indicated *MRR1* allele (heterozygous) with or without *MDR1* on YPD or YPD supplemented with 1 mM diamide or 6 mM H_2_O_2_. Similar results seen on two separate days. Strain names in bold font were shown to contain constitutively active Mrr1 variants and have high FLZ resistance (71). Plates were imaged after 24 or 48 h of growth at 37°C, as indicated. Download FIG S4, PDF file, 2.4 MB. Copyright © 2021 Demers et al. 2021 Demers et al. https://creativecommons.org/licenses/by/4.0/ This content is distributed under the terms of the Creative Commons Attribution 4.0 International license.

A screen of isogenic C. lusitaniae strains for growth in various concentrations of 48 chemical compounds resuspended from the Biolog Phenotype MicroArray microplates (Fig. S5) supported our findings that constitutive Mrr1 activity can increase sensitivity to oxidative stress. When comparing strains harboring either the low-activity Mrr1 ancestral variant or the constitutively active Mrr1-Y813C variant, with either *MDR1* intact or removed, we found there were minimal differences in growth in the medium used to resuspend the Biolog compounds (Fig. S5A), and many conditions caused less than a 25% difference in growth. Unsurprisingly, constitutive Mrr1 activity conferred Mdr1-dependent resistance to 12 compounds, including three triazoles (FLZ, propiconazole, and myclobutanil) (Fig. S5B and C). High Mrr1 activity also led to Mdr1-independent resistance to four additional compounds, including two additional azoles (3-amino-1,2,4-triazole and miconazole nitrate) (Fig. S5B and C). Eight compounds caused a largely Mdr1-independent decrease in growth in strains harboring the constitutively active Mrr1-Y813C variant: 6-azauracil, berberine, BAPTA, lithium chloride, aminacrine, sodium metasilicate, pentamidine isethionate, and potassium chromate (Fig. S5D). Though diverse, these compounds have been reported to have varied effects on microbial metabolism or respiration (47–53) and/or alter DNA/RNA integrity (54–59) directly or indirectly through oxidative damage. Interestingly, berberine and calcium inhibitors, such as BAPTA, have previously been shown to alter growth of antifungal-resistant *Candida* species (60–62). Strains lacking *MRR1* or harboring the low-activity Mrr1-L1191H+Q1197* variant were not as sensitive to some of these compounds, supporting our findings that secondary mutations causing a decrease or loss of Mrr1 activity can restore resistance in some oxidative stress environments (Fig. S5D).

10.1128/mBio.03328-20.5 FIG S5 Constitutive Mrr1 activity can negatively impact growth under a variety of conditions independent of *MDR1* expression. (A) Representative growth curve of U04 *mrr1*Δ strains complemented with the indicated *MRR1* allele in YPD over 16 h at 37°C; means ± SDs shown. (B) Heat map of relative growth in chemical compounds resuspended from Biolog phenotype MicroArray microplates after 16 h at 37°C for indicated U04 *mrr1*Δ complemented strains; growth of each strain was normalized to growth in YPD alone and then U04 *mrr1*Δ+*MRR1^ancestral^* for each condition and log_10_ transformed. Conditions in which neither *MRR1^Y813C^* expressing strain showed at least a 25% difference in growth compared to the strain expressing *MRR1^ancestral^* were excluded. Data shown were assayed in singlicate. (C) Subset of chemicals from panel B to which constitutive Mrr1 activity conferred an increase in growth. (D) Subset of chemicals from panel B to which constitutive Mrr1 activity conferred a decrease in growth that was not completely *MDR1* dependent. Download FIG S5, TIF file, 0.9 MB. Copyright © 2021 Demers et al. 2021 Demers et al. https://creativecommons.org/licenses/by/4.0/ This content is distributed under the terms of the Creative Commons Attribution 4.0 International license.

To gain insight into the mechanisms that lead to differences in oxidative stress resistance between strains with different levels of Mrr1 activity, we compared the gene expression profiles after a 30-min exposure to 0.5 mM H_2_O_2_, a partially inhibitory concentration. H_2_O_2_ exposure had broad strain-independent effects on the transcriptome, altering expression of 786 genes (fold change [FC] ≥ 2, false-discovery rate [FDR] < 0.05), including increased expression of *CLUG_04072*, a homolog of C. albicans catalase (*CAT1*), which was previously shown to be important for the resistance of C. lusitaniae to H_2_O_2_ (63) (see Fig. S6A and Table S2). While there were subtle differences in the H_2_O_2_ response between strains expressing the constitutively active Mrr1-Y813C variant compared to U04 *mrr1*Δ+*MRR1^ancestral^*, there were no clear patterns that would explain the difference in H_2_O_2_ resistance (Fig. S6B). The majority of differences in gene expression were seen in the magnitude of induction of Mrr1-regulated genes by H_2_O_2_, a known inducer of Mrr1 in other species (24, 32) (Fig. S6B and C; Table S2). We analyzed the expression of homologs of oxidative stress response (OSR) genes previously characterized in C. albicans (*Ca*) and Saccharomyces cerevisiae (*Sc*) and found that there was not a significant Mrr1-dependent difference in basal or H_2_O_2_-induced expression of these genes (Fig. S6A). Genes assessed included the OSR transcription factor encoded by *CaCAP1* or *ScYAP1*, superoxide dismutase (*SOD2*, *SOD4*, and *SOD6*), enzymes involved in the thioredoxin (*TSA1*, *TRX1*, and *TRR1*) and glutathione (*GPX* and *GSH1*) systems, catalases, and OSR genes involved in carbohydrate metabolism and the DNA-damage response (64, 65). Further analysis is required to better understand the link between constitutive Mrr1 activity and H_2_O_2_ sensitivity; however, these data highlight that the sensitivity is not due to failure to induce an oxidative stress response but is more likely a consequence of Mrr1-regulated genes, such as *MDR1*.

10.1128/mBio.03328-20.6 FIG S6 Constitutive Mrr1 activity alters how C. lusitaniae responds to H_2_O_2_ but not through classical oxidative stress response pathways. (A) Heat map of normalized CPMs following growth in 0.5 mM H_2_O_2_ for C. lusitaniae homologs of genes annotated as important for OSR in other fungal species, including C. albicans and S. cerevisiae; names of predicted C. albicans homologs listed. Strains used for RNA-Seq analysis include the unaltered U04 (*MRR1^Y813C^*), U04 *mrr1*Δ, and *MRR1* complemented strains in the U04 *mrr1*Δ background, which are denoted by their respective *MRR1* alleles. Hierarchical clustering of row (genes) and columns (strains) by Euclidean distance, no gene expression cutoffs or FDR filters applied. One replicate for U04 *mrr1*Δ and U04 *mrr1*Δ+*MRR1^ancestral^* in 0.5 mM H_2_O_2_ was excluded from the heat map but not statistical analyses. Allele names in bold font confer high Mrr1 activity. (B) Heat map of normalized CPMs for genes with a significant interaction between strains expressing *MRR1^Y813C^* and 0.5 mM H_2_O_2_ treatment compared to U04 *mrr1*Δ+*MRR1^ancestral^*. Allele names in bold font confer high Mrr1 activity; gene names in bold font were found to be Mrr1 regulated (Table S1). Hierarchical clustering of row (genes) by Euclidean distance. (C) Heat map of normalized CPMs for Mrr1-regulated genes as defined in Fig. 1B. Hierarchical clustering of row (genes) by Euclidean distance; gene names in bold font were found to have a significant interaction between strains expressing *MRR1^Y813C^* and 0.5 mM H_2_O_2_ treatment compared to U04 *mrr1*Δ+*MRR1^ancestral^*, shown in panel B. Allele names in bold font confer high Mrr1 activity. Download FIG S6, TIF file, 1.4 MB. Copyright © 2021 Demers et al. 2021 Demers et al. https://creativecommons.org/licenses/by/4.0/ This content is distributed under the terms of the Creative Commons Attribution 4.0 International license.

10.1128/mBio.03328-20.8 TABLE S2 RNA-Seq analysis of the interaction between Mrr1 activity and H_2_O_2_ response. Sheet A shows the interaction between constitutive Mrr1 activity and the response to H_2_O_2_; genes with a fold change of >2 (FDR < 0.05) are highlighted in dark red (upregulated) or blue (downregulated). Gene names in bold font were significant in both the U04 and U04 *mrr1*Δ+*MRR1^Y813C^* interaction. Sheet B shows the strain-independent response to H_2_O_2_. Sheet C shows the complete list of normalized CPMs used for the H_2_O_2_ linear model analysis. Download Table S2, XLSX file, 6.2 MB. Copyright © 2021 Demers et al. 2021 Demers et al. https://creativecommons.org/licenses/by/4.0/ This content is distributed under the terms of the Creative Commons Attribution 4.0 International license.

### Phenotype dynamics in chronic infection populations over time.

**FIG 5:**
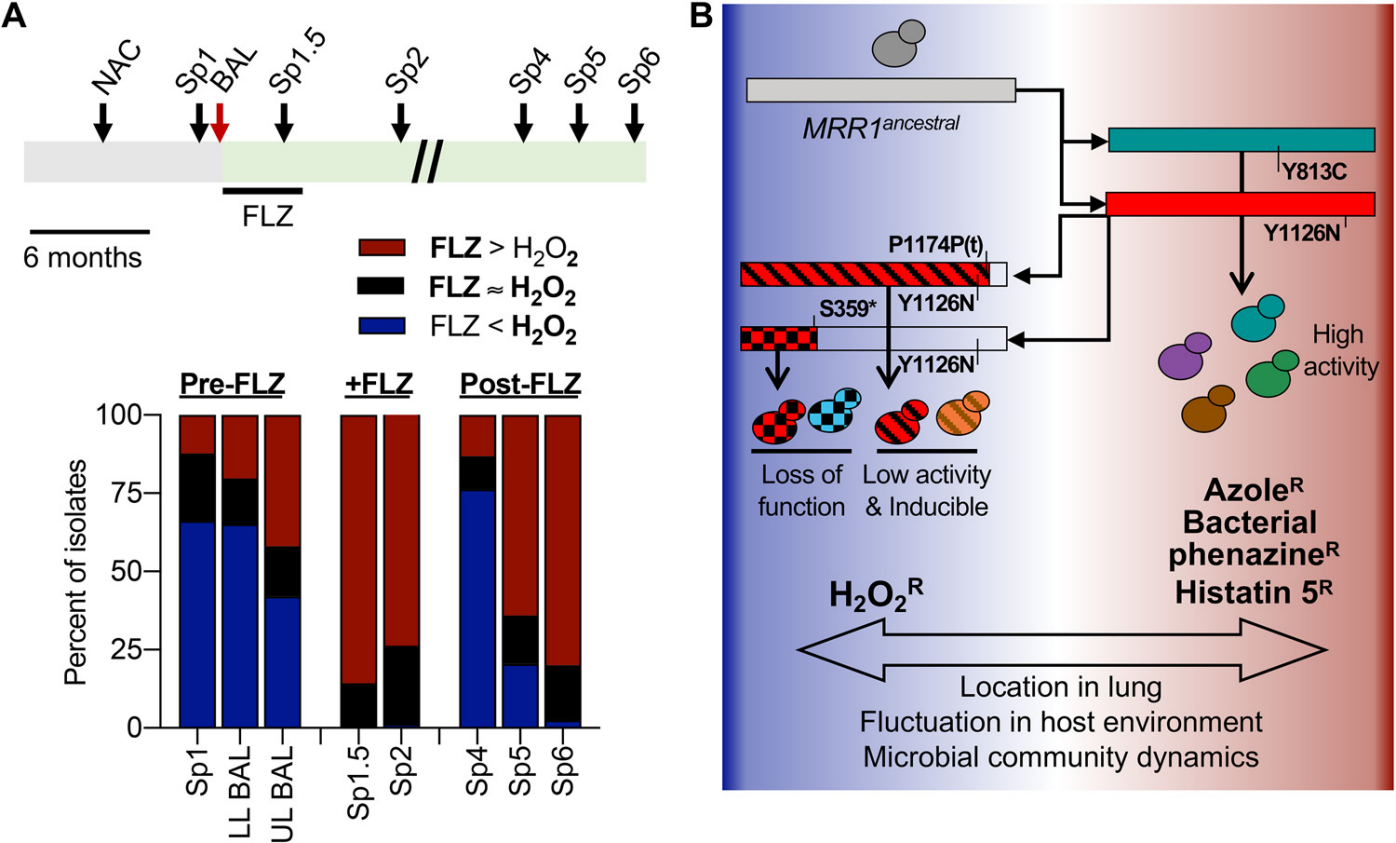
Trade-off between FLZ and H_2_O_2_ resistance persists in evolving C. lusitaniae populations during a chronic lung infection. (A) Schematic of sampling timeline (top) and histogram of the number of isolates that (i) were mostly uninhibited on FLZ, but were inhibited by H_2_O_2_ (red), (ii) were mostly uninhibited on H_2_O_2_ but were inhibited by FLZ (blue), or (iii) were uninhibited under both conditions (black). For the schematic, the gray bar represents the 6 to 10 months before the BAL during which this patient was identified as being colonized by non-*albicans Candida* (NAC) species. C. lusitaniae was determined to be the dominate microbe in the upper and lower lobe (UL and LL, respectively) BAL samples (red arrow), which marks the start of the green bar. Sp1 was obtained 1 month before the BAL and was retrospectively also found to contain abundant C. lusitaniae. Sp1.5, Sp2, Sp4, Sp5, and Sp6 were obtained 3, 9, 32, 35, and 38 months, respectively, after the BAL and all contained C. lusitaniae. A 4-month course of FLZ therapy was given after the BAL. Scale bar indicates 6 months. Multiple isolates were collected from each sample/timepoint (*n* = 38 to 80) and assayed for growth on YPD supplemented with 8 μg/ml FLZ or 4 mM H_2_O_2_. Growth was scored as completely inhibited, partially inhibited or uninhibited compared to that of a YPD-only control. (B) Model for the evolution of C. lusitaniae *MRR1* in this population. Whole-genome sequencing and mutation analyses suggest that following the initial infection with C. lusitaniae harboring the Mrr1-ancestral variant, a combination of exposure to different stimuli that changed overtime or by locations within the CF lung environment led to the selection for a heterogeneous population. Multiple constitutively active Mrr1 variants arose, and while some persisted over time, others were subsequently mutated again. The secondary mutations causing premature stop codons (represented by shortened bars) resulted in reversion to low Mrr1 activity that was inducible or complete loss of Mrr1 activity. The balance between selective pressures resulted in a heterogeneous population of isolates with varied resistance (^R^) to biologically and clinically important compounds.

In light of the evidence for complex evolution of *MRR1* and the potentially advantageous phenotypes associated with both high and low Mrr1 activity, we sought to better understand the fraction of isolates with these Mrr1-associated traits over time. For this analysis we used arrayed C. lusitaniae populations isolated from one BAL procedure sample or from sputum collected from the same subject over 3 years, with the first time point approximately 6 months after the first clinical culture report of the high levels of “non-*albicans Candida*” as shown in Fig. 5A. When screening isolates for growth on FLZ (8 μg/ml) or H_2_O_2_ (4 mM), we found an inverse correlation between growth on FLZ and growth on H_2_O_2_ (Fig. 5A). It was uncommon for isolates to be uninhibited under both conditions. Isolates from the early samples were predominately sensitive to FLZ (10) and resistant to H_2_O_2_. During and soon after the course of FLZ therapy (Sp1.5 and Sp2, respectively), however, there was an increase in isolates that were FLZ resistant and H_2_O_2_ sensitive (Fig. 5A). Subsequent samples from 2 years after the FLZ therapy was completed varied in the proportion of isolates that grew better on H_2_O_2_ and FLZ. Thus, over the course of 3 years, we repeatedly identified isolates that were either FLZ resistant and H_2_O_2_ sensitive, or vice versa, but not typically resistant to both, further supporting a trade-off between these phenotypes (Fig. 5B).

## DISCUSSION

A population of C. lusitaniae isolates first described by Demers et al. (10) contained an unexpectedly large number of nonsynonymous mutations in the gene encoding the transcription factor Mrr1, suggesting that Mrr1 activity was under strong selective pressure *in vivo*. These *MRR1* alleles contained either one or two nonsynonymous SNPs or indels (Fig. 1A), and isolates with one mutation had on average higher FLZ resistance than those with two nonsynonymous *MRR1* mutations (Fig. 2A). Deconstruction of *MRR1* alleles with two mutations revealed an evolutionary path in which an activating mutation arose first (e.g., Y1126N) and was followed by suppressing mutations, in the form of premature stop codons, that either restored low basal activity with retention of inducibility [e.g., P1174P(t)], or abolished Mrr1 activity altogether (S359*) (Fig. 3 and see Fig. S3 in the supplemental material). Our findings from the deconstruction of the *MRR1^**Y1126N**+P1174P(t)^* and MRR1^**Y1126N**+S359^* alleles (common mutation in bold) led us to propose that similar evolutionary processes likely occurred in lineages containing the L1191H and R1066S mutations (Fig. 1A). It is interesting to note that suppressor mutations only arose in alleles encoding activating mutations affecting the C terminus (Y1126N, L1191H, and R1066S) (see Fig. 2B) and not in alleles with activating mutations affecting the central regulatory domain (e.g., Y813C). This suggests that there may be functional differences between these two types of activating mutations that require future exploration or that these lineages experienced different local environments. Interestingly, a Candida parapsilosis strain was recently found to contain a central domain mutation and a premature stop codon (Mrr1^P295L+Q1074^*) similar to the alleles described above, suggesting that selection for and against elevated Mrr1 activity may also occur in other *Candida* species; however, it is not currently known how these individual mutations impact Mrr1 activity and FLZ resistance (66). While previous studies have identified GOF and LOF mutations in genes such as *MRR1*, *TAC1*, *PDR1*, *UPC2*, *ERG3* and *ERG11* that cause increased antifungal resistance (reviewed in references 67 and 68), few instances of sequential mutations in the same gene that cause first an increase then a decrease in activity have been described. Interestingly, multiple studies have shown that alteration in gene copy number, which can be achieved by partial to whole chromosome aneuploidy, can be selected for during stressful conditions and lost upon cessation of the stress, allowing for reversion to a “wild-type” phenotype (reviewed in references 68 and 69). These studies intriguingly parallel our own in that they show both positive and negative selection on a particular phenotype and can result in complex populations with mixed phenotypes. Demers et al. (10) characterized the aneuploidies present in the 20 clinical isolates utilized in these studies; thus far, gene copy number variation has not been determined to be a major driver of antifungal resistance in this C. lusitaniae population.

The RNA-Seq analysis of isogenic strains expressing different *MRR1* alleles was consistent with prior studies and showed that C. lusitaniae Mrr1 both positively and negatively regulates gene expression (Fig. 1D), although further analysis is required to determine which genes are direct targets of Mrr1. Adding to previous studies in C. lusitaniae (10, 29) and C. albicans (24), we found that Mrr1 positively regulates 41 genes with a fold change of ≥2 and 102 genes with a fold change of ≥1.5 (FDR < 0.05). Mrr1-induced genes include multiple MFS and ABC transporters (i.e., *MDR1*, *FLU1*, *CDR1*), methylglyoxal reductases (37), putative alcohol dehydrogenases, and a variety of other putative metabolic genes (Table S1). Constitutively active Mrr1 also appears to repress expression of 42 genes (fold change ≥ 1.5, FDR < 0.05), including multiple iron and/or copper transporters and reductases and sugar transporters (Table S1). These data combined with those from Biermann et al., who showed that C. lusitaniae Mrr1 is induced by the spontaneously formed stress signal methylglyoxal (37), imply that Mrr1 may play a larger role in a generalized metabolic or stress response beyond what has been previously studied in response to FLZ and xenobiotic stressors. We propose that potential metabolic differences may account for slight strain-to-strain variability in FLZ MIC when expressing the same *MRR1* variants, such as the subtle difference seen in the U04 and U05 backgrounds when expressing *MRR1^ancestral^* and MRR1^L1191H+Q1197^* (Fig. 1B and C).

The analysis of isogenic and nonisogenic strains showed that the C-terminal region of C. lusitaniae Mrr1 is necessary for constitutive Mrr1 activity but not required for induction of Mrr1-regulated genes, such as *MDR1* and *MGD1*, in response to benomyl (Fig. 2D, 3, and S3). Mutations resulting in the loss pf >200 amino acids, however, caused strains to phenocopy *mrr1*Δ strains (Fig. 2D and 3C and D). These data are consistent with previous studies showing C-terminal truncations prior to amino acid 944 in C. albicans *MRR1*, homologous to position 1116 in C. lusitaniae *MRR1*, caused a complete loss of C. albicans Mrr1 activity (34). Surprisingly, LOF Mrr1 variants and *mrr1*Δ strains showed intermediate expression of a subset of the most strongly differentially regulated genes compared to that of strains with low-activity Mrr1 (Fig. S2B), which has not been observed in other *Candida* species (24, 26, 28). Elevated *MDR1* expression in strains lacking functional Mrr1 caused the unexpectedly high FLZ resistance (Fig. S1A). Though not specifically noted, a slight increase in FLZ resistance was also reported by Kannan et al. (29) upon deletion of *MRR1* from their FLZ-sensitive C. lusitaniae isolate P1, supporting our conclusion that this phenomenon spans diverse C. lusitaniae isolates. Additional studies are required to determine if this phenomenon is unique to C. lusitaniae or is more broadly shared among non-*albicans Candida* species closely related to C. lusitaniae, such as C. auris (20, 70). Furthermore, while we have shown that the increase in *MDR1* expression in *mrr1*Δ strains is Cap1 independent, additional analyses are required to determine the involvement of other coregulators of the Mrr1 regulon previously described in C. albicans (32, 71, 72) and determine if these relationships are conserved in C. lusitaniae.

The repeated acquisition of a second mutation in alleles encoding constitutively active Mrr1 variants raised the question as to why, if constitutive Mrr1 was initially selected for, would it later be selected against *in vivo*. From previous studies, it is clear that constitutive Mrr1 activity can be beneficial under a variety of biologically relevant conditions, including exposure to azoles (10, 24), bacterium-produced toxins, including phenazines (10), and host-produced antifungal peptides, including histatin 5 (10, 39). However, little is known about why constitutively active Mrr1 variants would be selected against if there is not a growth defect (Fig. 4A and Fig. S5A). Here, we explored conditions relevant to a chronic lung infection, such as the one these isolates originated from (Fig. 4B). Chronic lung infections are typically an inflamed environment containing a high number of polymorphonuclear leukocytes (PMNs) that produce proteases, myeloperoxidases, and ROS (73, 74), which are important components of the immune system used to kill fungi (reviewed in reference 75). In a screen of diverse chemical compounds, we found that strains with constitutive Mrr1 activity were more strongly inhibited by multiple compounds that have previously been shown to cause damage through oxidative stress (Fig. S5B to D). When we specifically interrogated H_2_O_2_ resistance, we found that C. lusitaniae strains harboring constitutively active Mrr1 variants were more sensitive than strains harboring low-activity Mrr1 variants (Mrr1 ancestral and Mrr1 variants lacking <100 amino acids from the C terminus) or lacking a functional Mrr1 (Fig. 4A and S4). Sensitivity to H_2_O_2_ and the compounds from the Biolog plates was at least partially dependent on Mdr1, though other Mrr1-regulated genes, such as those involved in metabolism, may also contribute to the decreased growth under conditions of oxidative stress (Fig. S4B, S5, and S6). Interestingly, the trade-off between FLZ and H_2_O_2_ resistance was conserved in other *Candida* species and among a time series of C. lusitaniae isolates (Fig. 4C and 5A).

As outlined in the model in Fig. 5B, together, these data highlight that changing environments within complex and dynamic chronic infections could contribute to the development of heterogeneous fungal populations. Though it appears that the initial selection on the ancestral version of Mrr1 was driven by the need for increased Mrr1 activity, over time, either these selective pressures were removed or other pressures became dominant, resulting in a second mutation in some alleles. In most cases, this secondary wave of mutations caused a decrease or loss of Mrr1 activity that further contributed toward a population with mixed levels of FLZ resistance (Fig. 5B). Although the exact selective pressures at play in this instance are unknown, these data highlight the importance of understanding how microbes evolve *in vivo*, as complex environments, even in the absence of clinically used antifungals, can shape the microbial population and lead to antimicrobial resistance.

## MATERIALS AND METHODS

### Strains and growth conditions.

*Candida* strains used in this study are listed in Table S3 in the supplemental material. All strains were stored as frozen stocks with 25% glycerol at −80°C and subcultured on YPD (1% yeast extract, 2% peptone, 2% glucose, 1.5% agar) plates at 30°C. Strains were regularly grown in YPD liquid medium at 30°C on a roller drum. Cells were grown in YNB (0.67% yeast nitrogen base medium with ammonium sulfate [RPI Corp.]) liquid supplemented with either 2% glucose, 2% glycerol, or 2% Casamino Acids and in RPMI 1640 (Sigma; containing l-glutamine, 165 mM morpholinepropanesulfonic acid [MOPS], 2% glucose) liquid where noted. Medium was supplemented with 8 μg/ml FLZ (stock 4 mg/ml in dimethyl sulfoxide [DMSO]), 1 mM diamide (stock 58 mM in water), or 1 to 6 mM H_2_O_2_ (30% [wt/vol] in water, ∼9.8 M) where noted. Escherichia coli strains were grown in LB with either 100 μg/ml carbenicillin or 15 μg/ml gentamicin as necessary to obtain plasmids. BAL fluid and sputum were obtained in accordance with institutional review board protocols as described in reference 76.

10.1128/mBio.03328-20.9 TABLE S3 Strains used in this study. Download Table S3, DOCX file, 0.1 MB. Copyright © 2021 Demers et al. 2021 Demers et al. https://creativecommons.org/licenses/by/4.0/ This content is distributed under the terms of the Creative Commons Attribution 4.0 International license.

### DNA for gene knockout constructs.

Gene replacement constructs for knocking out *MRR1* (*CLUG_00542*, as annotated in reference 10), *MDR1* (*CLUG_01938/9*, as annotated in reference 10), and *CAP1* (*CLUG_02670*) were generated by fusion PCR as described by Grahl et al. (63). All primers (IDT) used are listed in Table S4. Briefly, 0.5 to 1.0 kb of the 5′ and 3′ regions flanking the gene was amplified from U04 DNA, isolated using the MasterPure yeast DNA purification kit (Epicentre). The codon-optimized nourseothricin (*NAT1* [77]) or hygromycin (*HygB*) resistance cassette was amplified from plasmids pNAT (78) and pYM70 (79), respectively, using the Zyppy plasmid miniprep kit (Zymo Research). Nested primers within the amplified flanking regions were used to stitch the flanks and resistance cassette together. PCR products for transformation were purified and concentrated with the Zymo DNA Clean & Concentrator kit (Zymo Research) with a final elution in molecular biology-grade water (Corning).

10.1128/mBio.03328-20.10 TABLE S4 Oligos and primers used in this study. Download Table S4, DOCX file, 0.1 MB. Copyright © 2021 Demers et al. 2021 Demers et al. https://creativecommons.org/licenses/by/4.0/ This content is distributed under the terms of the Creative Commons Attribution 4.0 International license.

### DNA for insertion of *NAT1* at neutral site in C. lusitaniae genome.

The approximately 4,000-bp genomic region between *CLUG_03302* and *CLUG_03303* on chromosome 4, which was not predicted to contain any genes or promoter regions, was targeted as a potentially neutral insertion site. To create plasmid DH3261 containing *NAT1* flanked by homology to this region of chromosome 4, approximately 1.0 kb of the flanking regions (positions 228,652 to 229,651 and 229,701 to 230,691) was amplified from U05 genomic DNA (gDNA). All primers (IDT) used are listed in Table S4. *NAT1* was amplified from pNAT (78). PCR products were purified, concentrated, and then assembled with the vector (pRS426 [80]) linearized with KpnI-HF and SalI-HF (New England BioLabs) and treated with the phosphatase rSAP (New England BioLabs) using the NEBuilder HiFi DNA assembly cloning kit (New England BioLabs). Assemblies were transformed into high-efficiency NEB 5-alpha competent E. coli (New England BioLabs). The *NAT1* insertion construct was isolated from DH3261 by digestion with KpnI-HF and SalI-HF (New England BioLabs).

### Plasmids for complementation of *MRR1*.

Plasmids for complementing *MRR1* were created as described by Biermann et al. (37). For naturally occurring *MRR1* alleles, we amplified (i) the *MRR1* gene and terminator with ∼1,150 bp upstream for homology from the appropriate strain’s genomic DNA, (ii) the selective marker, *HygB*, from pYM70 (79), and (iii) ∼950 bp downstream of *MRR1* for homology from genomic U05 (identical sequence for all relevant strains) using primers (IDT) listed in Table S4. PCR products were cleaned up using the Zymo DNA Clean & Concentrator kit (Zymo Research) and assembled into pMQ30 using the S. cerevisiae recombination technique as previously described (81). Plasmids created in S. cerevisiae were isolated using a yeast plasmid miniprep kit (Zymo Research) and transformed into high-efficiency NEB 5-alpha competent E. coli (New England BioLabs). E. coli containing pMQ30-derived plasmids were selected for on LB containing 15 μg/ml gentamicin. Plasmids from E. coli were isolated using a Zyppy plasmid miniprep kit (Zymo Research) and subsequently verified by Sanger sequencing at the Dartmouth College Genomics and Molecular Biology Shared Resources Core. pMQ30*^MRR1^* complementation plasmids were linearized with NotI-HF (New England BioLabs), cleaned up with the Zymo DNA Clean & Concentrator kit (Zymo Research), and eluted in molecular biology-grade water (Corning) before transformation of ∼2 μg into C. lusitaniae strain U04 *mrr1*Δ or U05 *mrr1*Δ, as described below.

The *MRR1^ancestral^* allele sequence was amplified from gDNA of a closely related C. lusitaniae isolate (B_L06) that has the same *MRR1* sequence but lacked any of the nonsynonymous mutations that varied among the population of C. lusitaniae isolates described here (Fig. 1A). This *MRR1* sequence contains multiple synonymous and nonsynonymous mutations in comparison with that of the reference strain, ATCC 42720 (82). Additional *MRR1* alleles were amplified from gDNA from U04 (*MRR1^Y813C^*), U05 (MRR1^L1191H+Q1197^*), U02 (*MRR1^Y1126N+P1174P(t)^*) and U06 (MRR1^S359^*^+Y1126N^). While making the pMQ30^MRR1-S359^*^+Y1126N^ plasmid, one clone was identified that lacked the S359* mutation, resulting in the pMQ30*^MRR1-Y1126N^* plasmid. To create additional *MRR1* alleles that were not identified within any C. lusitaniae isolates, pieces of *MRR1* were selectively removed and repaired with DNA either containing or lacking the desired mutation. Because the L1191H and Q1197* mutations were close together, an alternate strategy was used to separate these mutations. DNA fragments synthesized by IDT containing either the L1191H or Q1197* mutations alone (sequences in Table S4) were amplified then assembled with pMQ30^MRR1-L1191H+Q1197^* (linearized with PvuI-HF) using the NEBuilder HiFi DNA assembly cloning kit (New England BioLabs). To remove an unexpected nonsynonymous mutation in pMQ30^MRR1-Q1197^*, this plasmid was digesting with EcoNI and repaired with a piece of DNA amplified from U04 *mrr1*Δ+*MRR1^ancestral^* lacking the unwanted mutation.

### Strain construction.

Mutants were constructed as previously described by Grahl et al. using an expression-free ribonucleoprotein CRISPR-Cas9 method (63). Briefly, 1 to 2 μg of DNA for gene knockout constructs generated by PCR or 2 μg of digested plasmid, purified, and concentrated with a final elution in molecular biology-grade water (Corning) was used per transformation. Plasmids containing complementation and knockout constructs and resulting strains are listed in Table S3 and CRISPR RNAs (crRNAs; IDT) are listed in Table S4. Transformants were selected on YPD agar containing 200 μg/ml nourseothricin or 600 μg/ml hygromycin B.

### Drug susceptibility assays.

MIC was determined using a broth microdilution method as previously described (83) with slight modifications (10). Briefly, 2 × 10^3^ cells were added to a 2-fold dilution series of FLZ prepared in RPMI 1640 medium, testing concentrations from 64 to 0.125 μg/ml, and then incubated at 35°C for 24 h. The MIC was defined as the drug concentration that abolished visible growth compared to that of a drug-free control.

### Quantitative RT-PCR.

Overnight cultures were back diluted to an optical density at 600 nm (OD_600_) of ∼0.1 and grown for 6 h in YPD liquid medium at 30°C. Then, 50 μg/ml of benomyl (stock 10 mg/ml in DMSO) or an equivalent volume of DMSO was added for experiments assessing the induction of Mrr1 activity; 7.5 μg RNA (harvested using the MasterPure yeast RNA purification kit [Epicentre]) was DNase treated with the Turbo DNA-free kit (Invitrogen). cDNA was synthesized from 300 to 500 ng of DNase-treated RNA using the RevertAid H Minus first-strand cDNA synthesis kit (Thermo Scientific), according to the manufacturer’s instructions for random hexamer primer (IDT) and a GC-rich template. Quantitative RT-PCR was performed on a CFX96 real-time system (Bio-Rad), using SsoFast Evergreen supermix (Bio-Rad) with the primers listed in Table S4. Thermocycler conditions were as follows: 95°C for 30 s and 40 cycles of 95°C for 5 s, 65°C for 3 s, and 95°C for 5 s. Transcripts were normalized to *ACT1* expression.

### RNA sequencing.

Overnight cultures were back diluted into YPD and grown to exponential phase (∼8 h) twice and then treated with vehicle or 0.5 mM H_2_O_2_ for 30 min, in biological triplicates. RNA was harvested from snap-frozen pellets (using liquid nitrogen) using the MasterPure yeast RNA purification kit (Epicentre) and stored at −80°C. RNA libraries were prepared using the Kapa mRNA HyperPrep kit (Roche) and sequenced using single-end 75-bp reads on the Illumina NextSeq 500 platform. The data analysis pipeline is available from the github repository (https://github.com/stajichlab/RNASeq_Clusitaniae_MRR1 and archived as https://www.doi.org/10.5281/zenodo.4477474). FASTQ files were aligned to the ATCC 42720 (82) genome with the splice-site aware and SNP-tolerant short-read aligner GSNAP (v v2019-09-12) (84). The alignments were converted to sorted BAM files with Picard (v2.18.3; https://broadinstitute.github.io/picard/), and read counts were computed with featureCounts (v1.6.2) (85) with updated genome annotation to correct a truncated gene model for locus *CLUG_00542* and combine a single gene split into two, *CLUG_01938_1939*; the reasoning for these changes is explained in reference 10. Differential gene expression analyses were performed with the edgeR (86) package in Bioconductor by fitting a negative binomial linear model. The resulting *P* values were corrected for multiple testing with the Benjamini-Hochberg procedure to control the false-discovery rate. Genes for which there were less than 2 counts per million (CPM) across the three (absent genes) were not included for differentially expressed gene analysis. Two separate linear models, described below, were created to define the Mrr1 regulon under control conditions alone and determine the interaction between Mrr1 activity and H_2_O_2_ exposure. Heat maps show normalized CPM values that are centered and scaled by gene and hierarchically clustered (Euclidean distance) using pheatmap (87).

To define the Mrr1 regulon in YPD alone, we identified genes differentially expressed between strains with constitutive Mrr1 activity (U04 and U04 *mrr1*Δ+*MRR1^Y813C^*) and low or no Mrr1 activity (U04 *mrr1*Δ, U04 *mrr1*Δ+*MRR1^ancestral^*, and U04 *mrr1*Δ+MRR1^L1191H+Q1197^*); this model contained 5,474 genes. We discarded genes for which (i) the log_2_FC was not greater than 1 (2-fold) (see Fig. 1B) or 0.585 (1.5-fold) (see Table S1) with an FDR of <0.05, (ii) the average CPM for replicates was not greater than 10 for any strain, and (iii) expression in both U04 and U04 *mrr1*Δ+*MRR1^Y813C^* was not similar. Results are summarized in Table S1, including the Mrr1 regulon as defined here (Table S1A) and the normalized CPM/gene used for this linear model (Table S1B).

To determine how constitutive Mrr1 activity impacted the response to H_2_O_2_, we identified the overlap between the interaction between U04 or U04 *mrr1*Δ+*MRR1^Y813C^* and exposure to 0.5 mM H_2_O_2_, compared to the reference strain (U04 *mrr1*Δ+*MRR1^ancestral^*) and condition (YPD alone); this model contained 5,600 genes. Results are summarized in Table S2, including the interaction between strains with constitutively active Mrr1 and H_2_O_2_ (Table S2A), the effect of H_2_O_2_ treatment (Table S2B), and all normalized CPM/gene used for this linear model (Table S2C). One U04 *mrr1*Δ and one U04 *mrr1*Δ+*MRR1^ancestral^* replicate from the 0.5 mM H_2_O_2_ treatment condition were excluded from heat maps but not statistical analyses, because they displayed signatures not congruent with the rest of the data and did not cluster with other replicates from those strains.

### Biolog phenotype MicroArray analysis.

The chemicals in Biolog plates PM22D and PM24C were resuspended in 100 μl YPD liquid and transferred to a sterile 96-well polystyrene plate (Fisher). One hundred microliters of cells adjusted to an OD of 0.01 in YPD was added to each well. Plates were incubated at 37°C for 24 h. A control plate containing no drug was grown simultaneously for comparison.

### Plate-based chemical sensitivity assays.

**(i) Serial dilution assays.** Following growth in YPD medium overnight with aeration at 30°C, cells were washed and diluted in water to an OD_600_ of 1. Serial dilutions of 10-fold were carried out in a microtiter plate to yield six concentrations ranging from approximately 10^7^ cells/ml (for OD_600_ of 1) to approximately 10^2^ cells/ml. Five microliters of each dilution was applied to YPD plates containing the designated concentration of H_2_O_2_, FLZ, or diamide. Images were captured after incubation at 37°C for 24 or 48 h.

### (ii) C. lusitaniae population screen.

Individual isolates were collected from BAL fluid or sputum from the same subject over the course of multiple years; isolates from each sample were saved in a 96-well array format. Isolates were grown in YPD overnight and then transferred to a 384-well plate, with four wells representing each individual isolate. Cultures were spotted onto YPD or YPD supplemented with 8 μg/ml FLZ or 4 mM H_2_O_2_ using a 384-pin replicator; the screen performed in singlicate. Plates were incubated for 48 h at 37°C. Growth was scored by eye as completely inhibited, partially inhibited, or uninhibited relative to growth on the YPD-only control.

### Luminex analysis.

Cytokines in BAL fluid samples were measured (pg/ml) in singlicate by Luminex using a Millipore human cytokine multiplex kit (EMD Millipore Corporation, Billerica, MA) according to the manufacturer’s instructions. Assays were performed by the DartLab–Immune Monitoring and Flow Cytometry Resource core at Dartmouth.

### Statistical analyses.

Statistical analyses were performed using GraphPad Prism 9.0.0 (GraphPad Software). Unpaired Student’s *t* tests (two-tailed) with Welch’s correction were used to evaluate the difference in FLZ MICs between isolates containing one of two mutations in *MRR1*. One and two-way analysis of variance (ANOVA) tests were performed across multiple samples with either Tukey’s multiple-comparison test for unpaired analyses or Sidak’s multiple-comparison test for paired analyses. *P* values of <0.05 were considered to be significant for all analyses performed and are indicated with asterisks or letters in the text as follows: *, *P* < 0.05; **, *P* < 0.01; ***, *P* < 0.001; ****, *P* < 0.0001. In figures where the statistics are indicated by lowercase letters, samples marked by the same lowercase letter are not significantly different from each other, and samples marked with different lowercase letters are significantly different, as detailed in the figure legends.

### Data availability.

The data supporting the findings in this study are available within the paper and its supplemental material and are also available from the corresponding author upon request. Whole-genome sequences for strains in Fig. 1A were previously published by Demers et al. (10) and can be found in NCBI under BioProject PRJNA433226. The sequence for *MRR1^ancestral^*, Sanger sequenced from isolate B_L06, is available in GenBank (MW553730). The raw sequence reads from the RNA-Seq analysis have been deposited into NCBI sequence read archive under BioProject PRJNA680763. Raw and processed RNA-Seq count data are available in Gene Expression Omnibus (GSE162151) and include minor updates to the genome annotation and assembly for C. lusitaniae.
