# Supplementary figures and images for: Balancing Positive and Negative Selection: In Vivo Evolution of Candida lusitaniae MRR1
Source: mBio. 2021 Mar 30;12(2):e03328-20. doi: 10.1128/mBio.03328-20 (PMC8092287; doi:10.1128/mBio.03328-20)

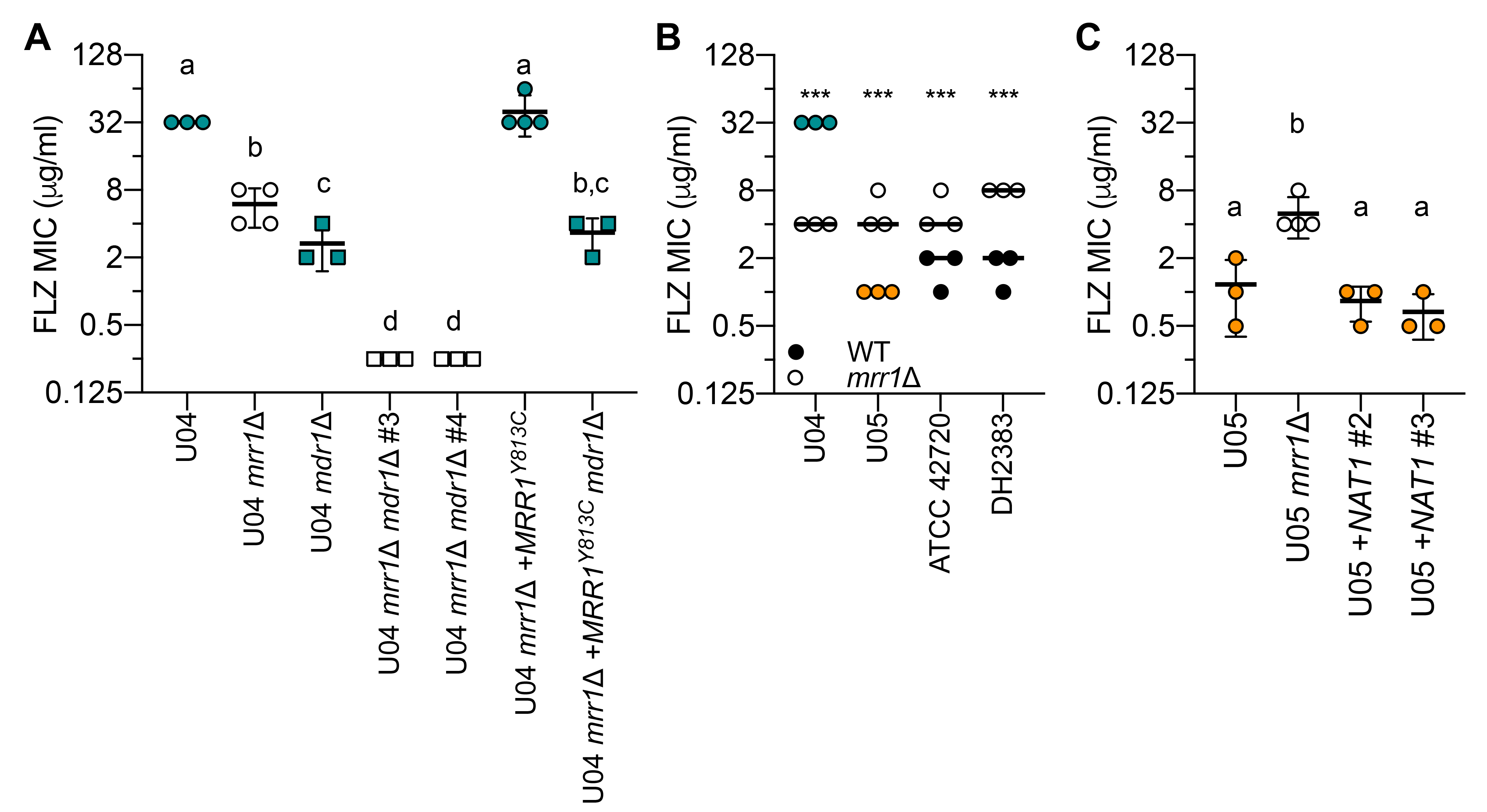

Supplement: FIG S1 [file mBio.03328-20-sf001.tif]

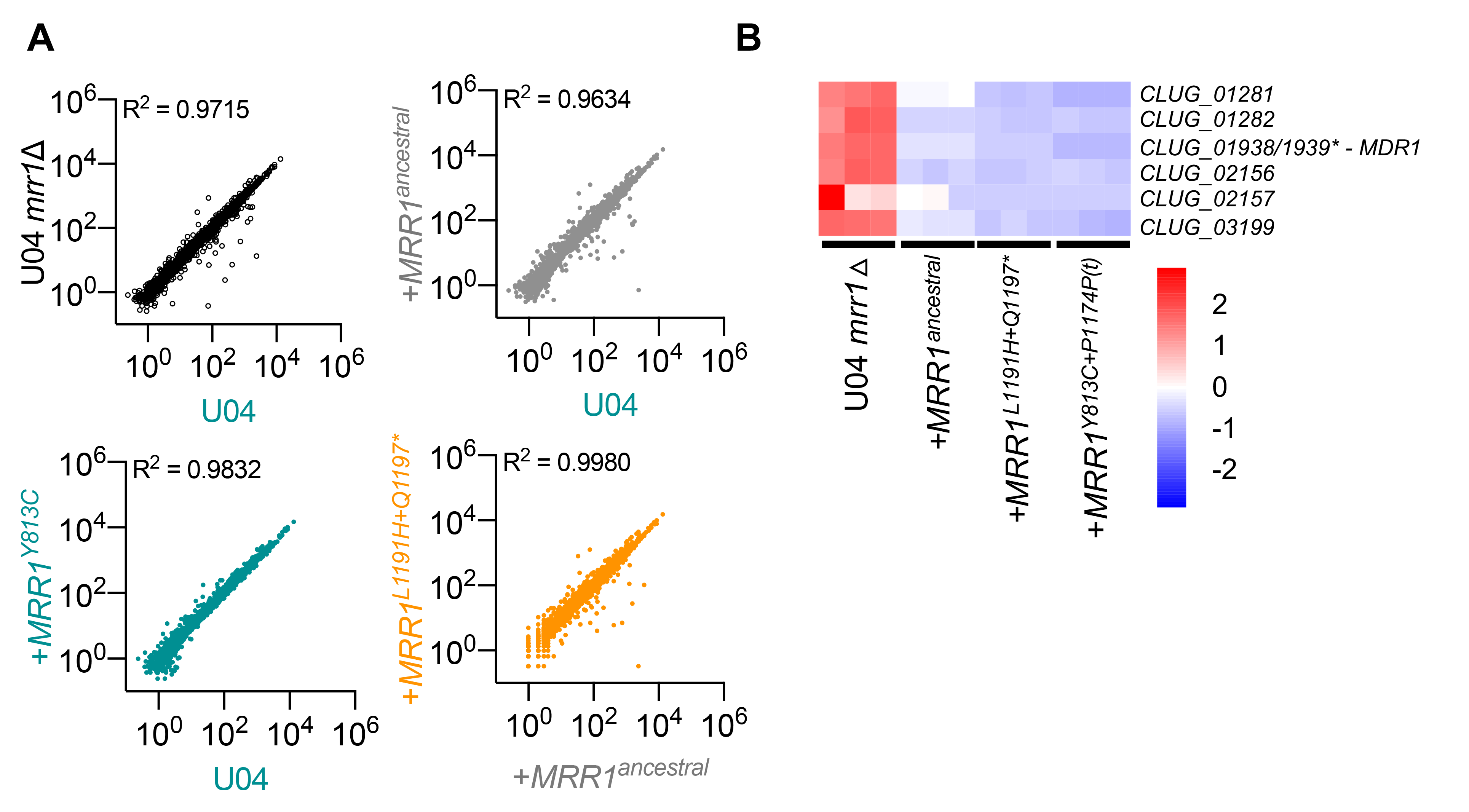

Supplement: FIG S2 [file mBio.03328-20-sf002.tif]

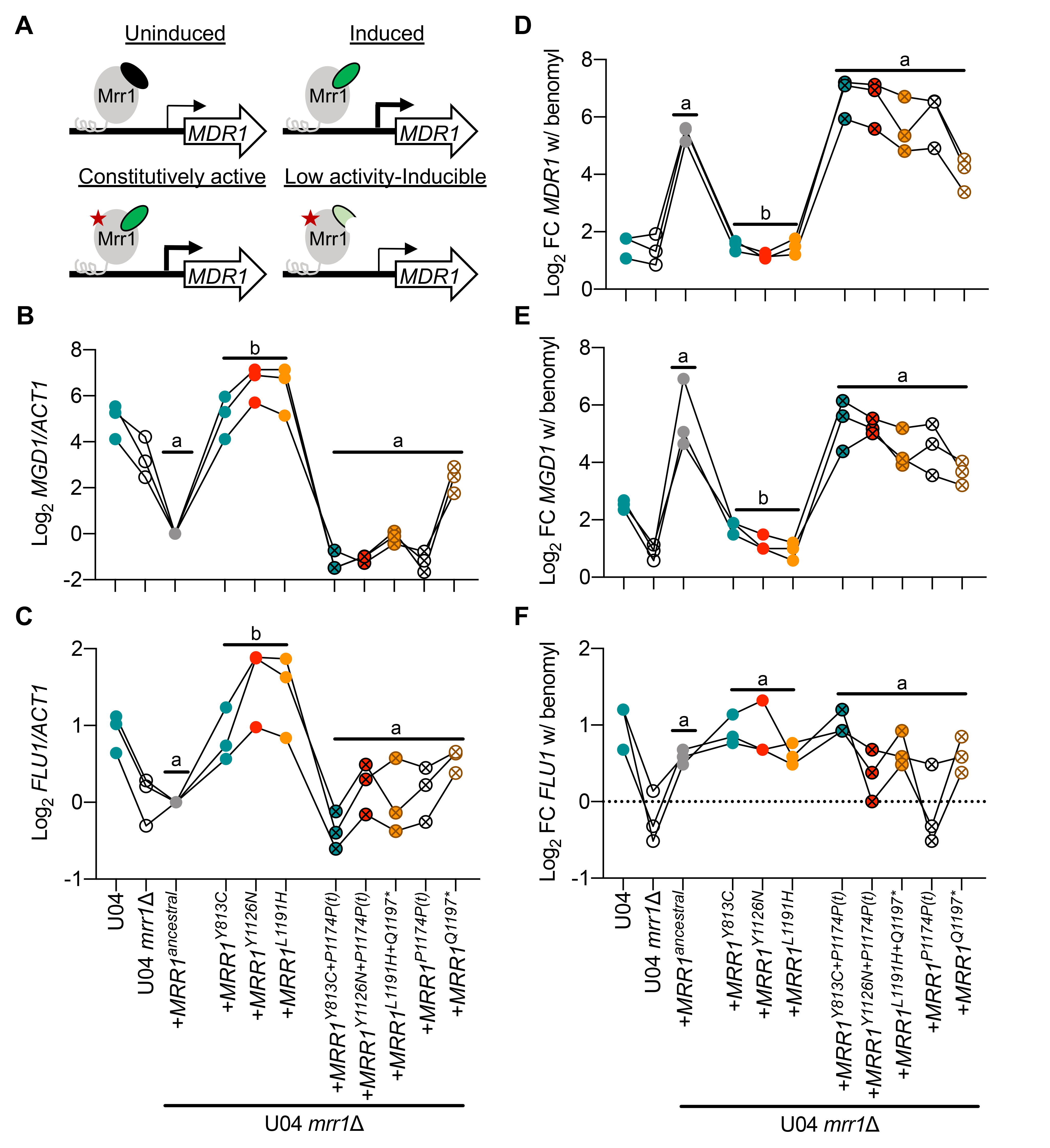

Supplement: FIG S3 [file mBio.03328-20-sf003.tif]

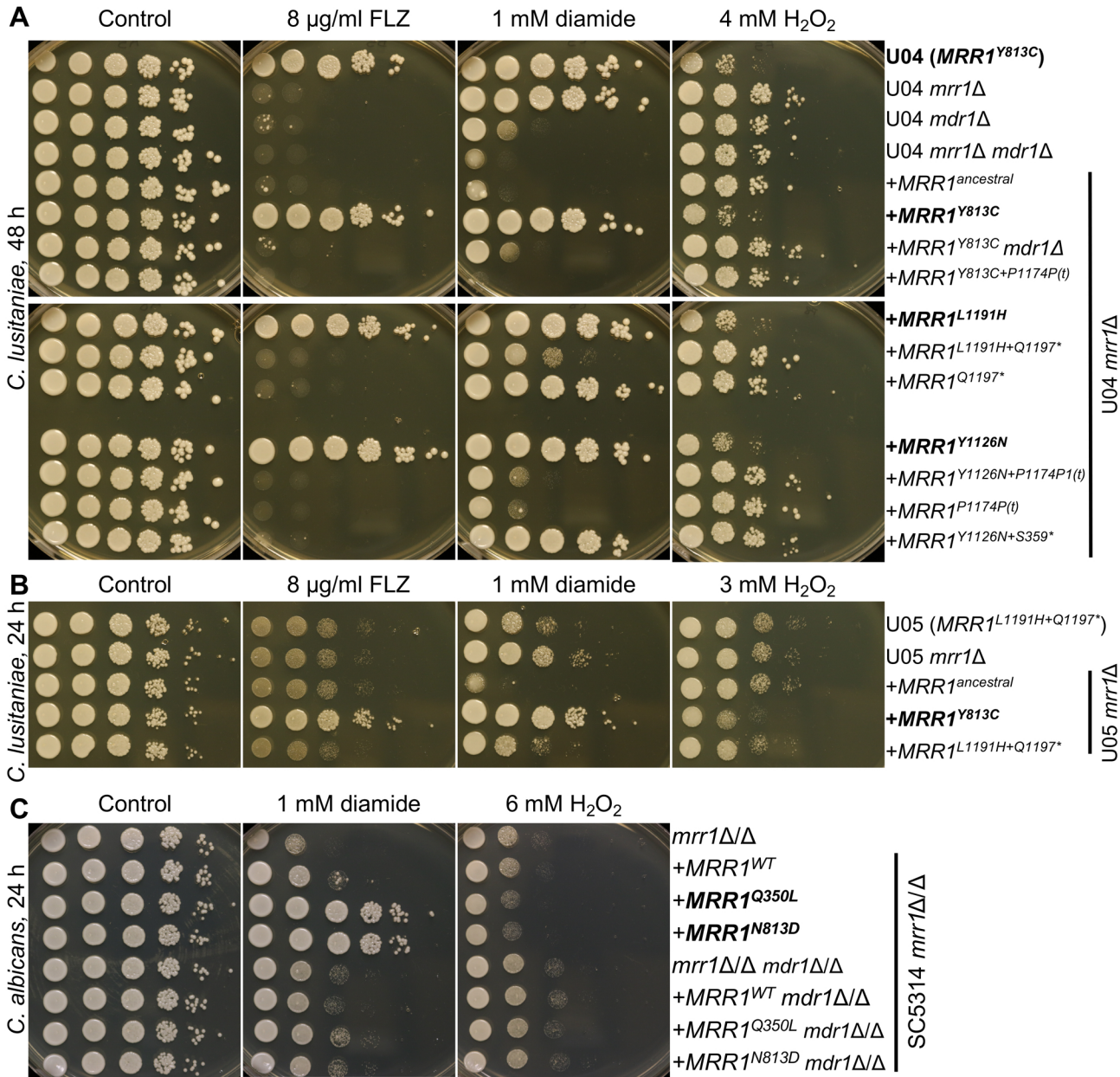

Supplement: FIG S4 [file mBio.03328-20-sf004.pdf]

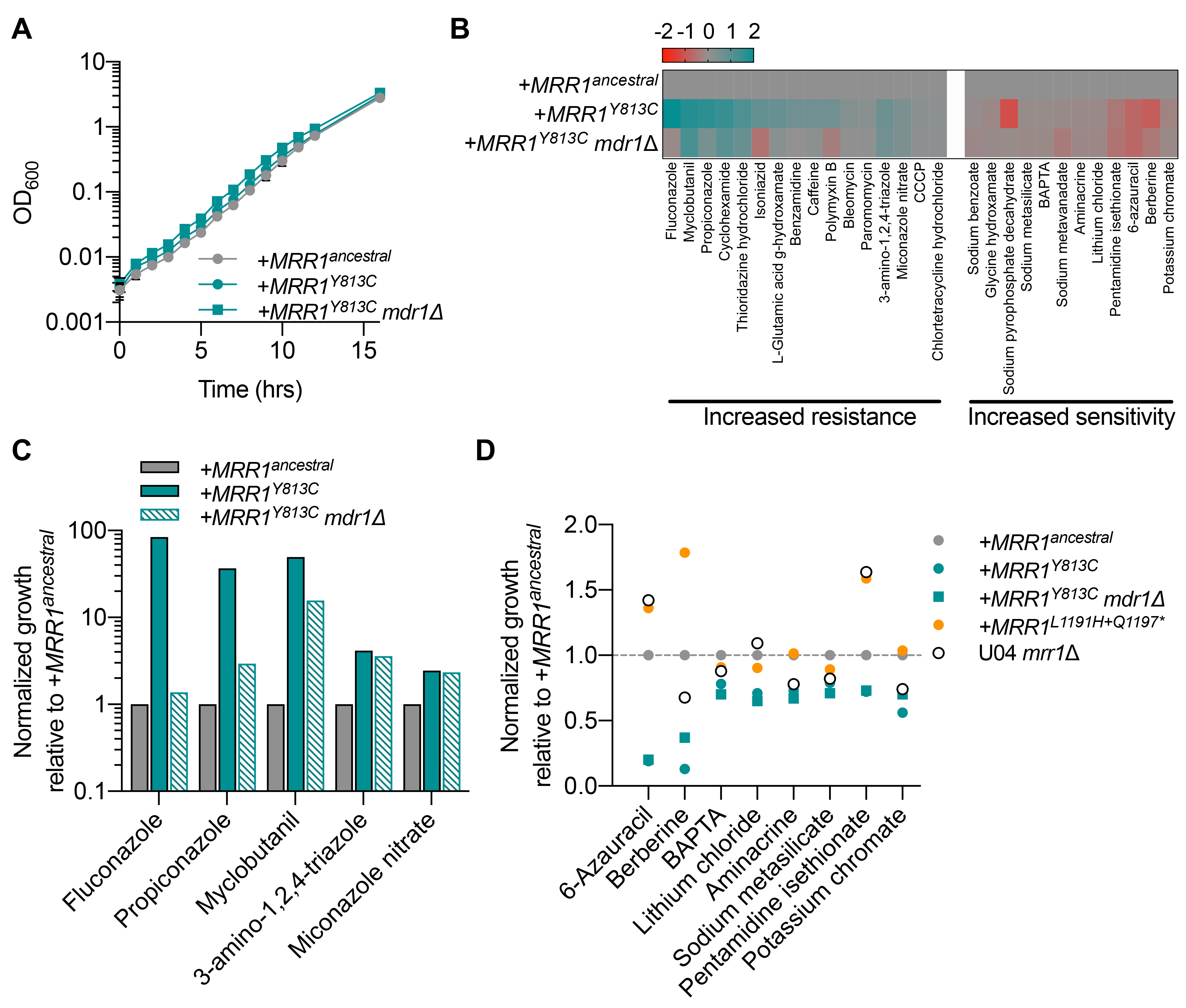

Supplement: FIG S5 [file mBio.03328-20-sf005.tif]

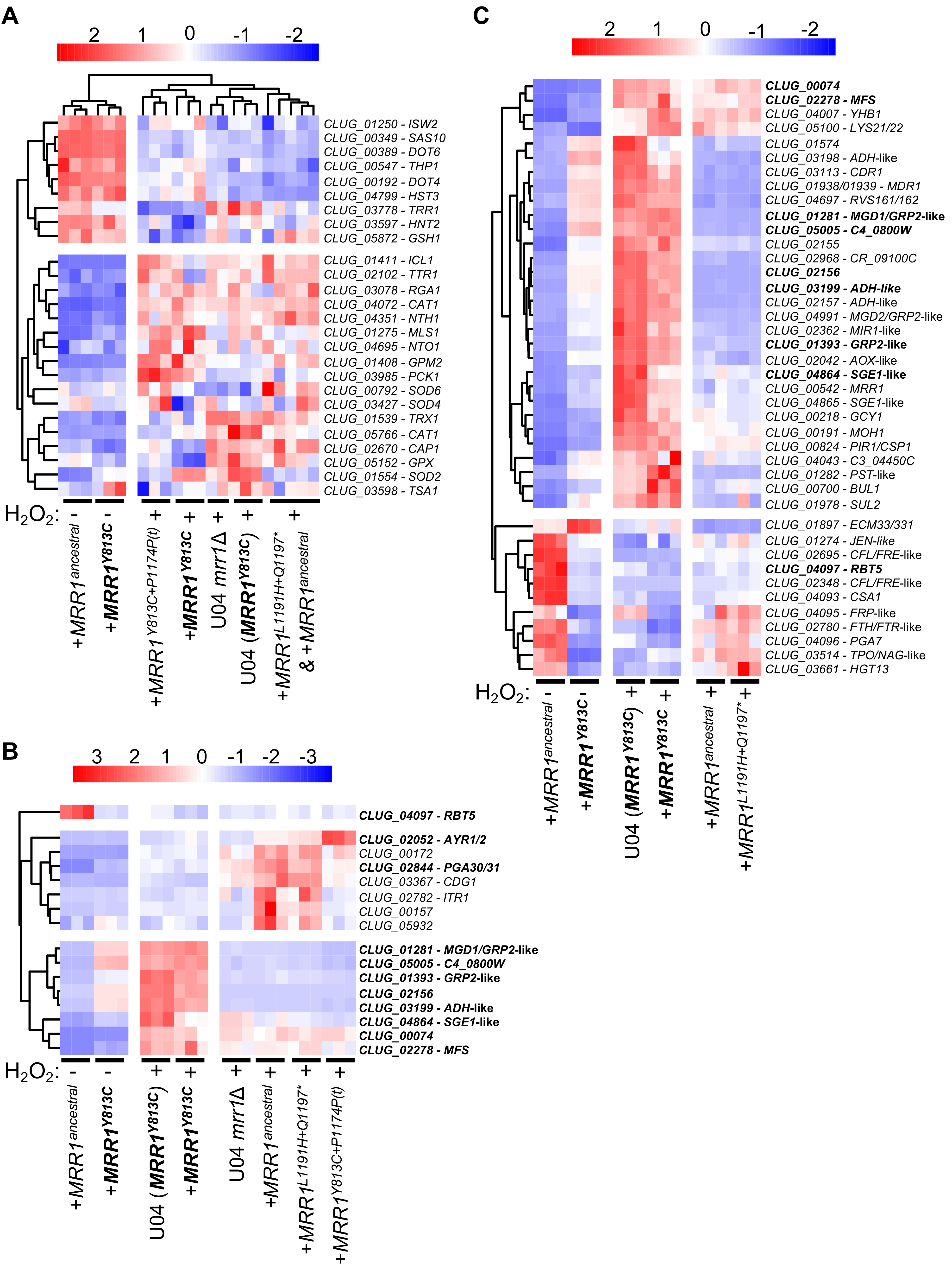

Supplement: FIG S6 [file mBio.03328-20-sf006.tif]
