# Supplementary material for: Balancing Positive and Negative Selection: In Vivo Evolution of Candida lusitaniae MRR1
Source: mBio. 2021 Mar 30;12(2):e03328-20. doi: 10.1128/mBio.03328-20 (PMC8092287; doi:10.1128/mBio.03328-20)
Supplement: TABLE S3 [file mBio.03328-20-st003.docx]

| Table S3. Strains used in this study. | | | | | | |  |  |
| --- | --- | --- | --- | --- | --- | --- | --- | --- |
| Strain | Lab # | Relevant genotype and information | Parental isolate | | | Source | |  |
| *C. lusitaniae* | | | | | | |  |  |
| U04 (A04) | DH2949 | *MRR1^Y813C^* |  | | | (1, 2) | |  |
| U04 *mrr1*Δ | DH3306 | *mrr1*Δ*::NAT1* | DH2949 | | | (2) | |  |
| U04 *mdr1*Δ | DH3112 | *mdr1*Δ::*HygB* | DH2949 | | | (2) | |  |
| U04 *mrr1*Δ *mdr1*Δ *#3* | DH3699 | *mrr1*Δ::*NAT1, mdr1*Δ::*HygB* | DH3306 | | | This study | |  |
| U04 *mrr1*Δ *mdr1*Δ *#4* | DH3700 | *mrr1*Δ::*NAT1, mdr1*Δ::*HygB* | DH3306 | | | This study | |  |
| U04 *mrr1*Δ *+MRR1^ancestral^* | DH3611 | *MRR1ancestral-HygB* | DH3306 | | | This study | |  |
| U04 *mrr1*Δ *+MRR1^Y813C^* | DH3613 | *MRR1^Y813C^-HygB* | DH3306 | | | (3) | |  |
| U04 *mrr1*Δ *+MRR1^Y813C+P1174P(t)^* | DH3616 | *MRR1^Y813C+P1174P(t)^-HygB* | DH3306 | | | This study | |  |
| U04 *mrr1*Δ *+MRR1^Y1126N^* | DH3618 | *MRR1^Y1126N^-HygB* | DH3306 | | | This study | |  |
| U04 *mrr1*Δ *+MRR1^Y1126N+P1174P(t)^* | DH3620 | *MRR1^Y1126N+P1174P(t)^-HygB* | DH3306 | | | This study | |  |
| U04 *mrr1*Δ *+MRR1^S359*+Y1126N^* | DH3622 | *MRR1^S359*+Y1126N^-HygB* | DH3306 | | | This study | |  |
| U04 *mrr1*Δ *+MRR1^P1174P(t)^* | DH3624 | *MRR1^Y1126N+P1174P(t)^-HygB* | DH3306 | | | This study | |  |
| U04 *mrr1*Δ *+MRR1^L1191H^* | DH3630 | *MRR1^L1191H^-HygB* | DH3306 | | | This study | |  |
| U04 *mrr1*Δ *+MRR1^Q1197*^* | DH3632 | *MRR1^Q1197*^-HygB* | DH3306 | | | This study | |  |
| U04 *mrr1*Δ *+MRR1^L1191H+Q1197*^* | DH3628 | *MRR1^L1191H+Q1197*^-HygB* | DH3306 | | | (3) | |  |
| U04 *mrr1*Δ *+MRR1^Y813C^ mdr1*Δ | DH3615 | *MRR1^Y813C^*::*HygB, mdr1*Δ::*NAT1* | DH3613 | | | This study | |  |
| U05 | DH3087 | *mrr1^L1191h+q1197*^* |  | | | (2) | |  |
| U05 *mrr1*Δ | DH3111 | *mrr1*Δ::*NAT1* | DH3087 | | | This study | |  |
| U05 *mrr1*Δ *cap1*Δ | DH3702 | *mrr1*Δ::*NAT1, cap1*Δ::*HygB* | DH3111 | | | This study | |  |
| U05 *mrr1*Δ *+MRR1^ancestral^* | DH3642 | *MRR1^ancestral^-HygB* | DH3111 | | | This study | |  |
| U05 *mrr1*Δ *+MRR1^Y813C^* | DH3646 | *MRR1^Y813C^-HygB* | DH3111 | | | This study | |  |
| U05 *mrr1*Δ *+MRR1^L1191H+Q1197*^* | DH3644 | *MRR1^L1191H+Q1197*^-HygB* | DH3111 | | | This study | |  |
| U05 +*NAT1* #2 | DH3634 | *NAT1-neutral site*, clone 2 | DH3087 | | | This study | |  |
| U05 +*NAT1* #3 | DH3635 | *NAT1-neutral site*, clone 3 | DH3087 | | | This study | |  |
| U06 | DH3100 | *MRR1^S359*+Y1126N^* |  | | | (2) | |  |
| U07 | DH3098 | *MRR1^Y1126N+P1174P(t)^* |  | | | (2) | |  |
| U08 | DH3091 | *MRR1^E722K^* |  | | | (2) | |  |
| L10 | DH3091 | *MRR1^Y813N^* |  | | | (2) | |  |
| L11 | DH3093 | *MRR1^R1066S+K912N(t)^* |  | | | (2) | |  |
| L12 | DH3094 | *MRR1^R1066S+Y1061*^* |  | | | (2) | |  |
| L17 | DH3101 | *MRR1^H467L^* |  | | | (2) | |  |
| ATCC 42720 | DH2387 |  |  | | | (4) | |  |
| ATCC 42720 *mrr1*Δ | DH3639 | *mrr1*Δ::*NAT1* | DH2387 | | | This study | |  |
| 2383 | DH2383 | *ura3*Δ, also known as RSY284/CL6936 |  | | | (5) | |  |
| 2383 *mrr1*Δ | DH3641 | *ura3*Δ*, mrr1*Δ::*NAT1* | DH2383 | | | This study | |  |
| B_L06 | DH3844 | *MRR1^ancestral^* |  | | | DHMC* | |  |
|  |  |  |  | | |  | |  |
| *C. albicans* |  |  |  | | |  | |  |
| SC5314 | DH3560 | Wild-type model strain, FLZ-sensitive |  | | | (6) | |  |
| SCMRR1M4A | DH3561 | *mrr1*Δ::*FRT*/*mrr1*Δ::*FRT* | SCMRR1M3A | | | (6) | |  |
| SCMRR1R44A | DH3567 | *MRR1*^G997V^-*FRT*/*MRR1*^G997V^-*FRT* | SCMRR1R43A | | | (7) | |  |
| SCMRR1R44C | DH3568 | *MRR1*^G997V^-*FRT*/*MRR1*^G997V^-*FRT* | SCMRR1R43C | | | (7) | |  |
| SCMRR1R84A | DH3569 | *MRR1*^T360I^-*FRT*/*MRR1*^T360I^-*FRT* | SCMRR1R83A | | | (7) | |  |
| SCMRR1R84B | DH3570 | *MRR1*^T360I^-*FRT*/*MRR1*^T360I^-*FRT* | SCMRR1R83B | | | (7) | |  |
| SCMRR1R94A | DH3571 | *MRR1*^K335N^-*FRT*/*MRR1*^K335N^-*FRT* | SCMRR1R93A | | | (7) | |  |
| SCMRR1R94B | DH3572 | *MRR1*^K335N^-*FRT*/*MRR1*^K335N^-*FRT* | SCMRR1R93B | | | (7) | |  |
| yLM417 |  | *mrr1*Δ::FRT/*mrr1*::P*_MRR1_*-*MRR1_WT_-SAT1* | SCMRR1M4A | | | (8) | |  |
| yLM418 |  | *mrr1*Δ::FRT/*mrr1*::P*_MRR1_*-*MRR1^Q350L^-SAT1* | SCMRR1M4A | | | (8) | |  |
| yLM420 |  | *mrr1*Δ::FRT/*mrr1*::P*_MRR1_*-*MRR1^N803D^-SAT1* | SCMRR1M4A | | | (8) | |  |
| yLM618 |  | *mrr1*Δ::FRT/*mrr1*Δ::FRT *mdr1*Δ::FRT/*mdr1*Δ::FRT | SCMRR1M4A | | | (8) | |  |
| yLM623 |  | *mrr1*Δ::FRT/*mrr1*::P*_MRR1_*-*MRR1^WT^-SAT1* *mdr1*Δ::FRT/*mdr1*Δ::FRT | yLM618 | | | (8) | |  |
| yLM624 |  | *mrr1*Δ::FRT/*mrr1*::P*_MRR1_*-*MRR1^Q350L^-SAT1* *mdr1*Δ::FRT/*mdr1*Δ::FRT | yLM618 | | | (8) | |  |
| yLM625 |  | *mrr1*Δ::FRT/*mrr1*:: P*_MRR1_* -*MRR1^N803D^ -SAT1* *mdr1*Δ::FRT/*mdr1*Δ::FRT | yLM618 | | | (8) | |  |
|  |  |  |  | | |  | |  |
| *C. dubliniensis* |  |  |  | | |  | |  |
| CM1 | DH3575 | FLZ-sensitive, from patient 1 |  | | | (9, 10) | |  |
| CM2 | DH3576 | FLZ-resistant, from patient 1 |  | | | (9, 10) | |  |
| CD57 | DH3577 | FLZ-sensitive |  | | | (10, 11) | |  |
| CD57A | DH3578 | FLZ-resistant, generated *in vitro* | CD57 | | | (10, 11) | |  |
| CD57B | DH3579 | FLZ-resistant, generated *in vitro* | CD57 | | | (10, 11) | | |
| CD51-II | DH3580 | FLZ-sensitive |  | | | (10, 11) | |  |
| CD51-IIA | DH3581 | FLZ-resistant, generated *in vitro* | CD51-II | | | (10, 11) | |  |
| CD51-IIB | DH3582 | FLZ-resistant, generated *in vitro* | CD51-II | | | (10, 11) | |  |
|  |  |  |  | | |  | |  |
| *S. cerevisiae* |  |  |  | | |  | |  |
| Cloning yeast | JH27 | For cloning |  | | |  | |  |
|  |  |  |  | | |  | |  |
| Plasmids in *E. coli* (DH5⍺) |  |  |  | | |  | |  |
| pMQ30*^MRR1-L1191H+Q1197*^* | DH3829 | pMQ30-*MRR1^L1191H+Q1197*^-HygB* complementation, Gent^R^ | | |  | (3) | |  |
| pMQ30*^MRR1-ancestral^* | DH3830 | pMQ30-*MRR1^ancestral^- HygB* complementation, Gent^R^ | | |  | This study | |  |
| pMQ30*^MRR1-Y813C^* | DH3831 | pMQ30-*MRR1^Y813C^- HygB* complementation, Gent^R^ | | |  | (3) | |  |
| pMQ30*^MRR1-Y813C+P1174P(t)^* | DH3832 | pMQ30-*MRR1^Y813C+P1174P(t)^-HygB* complementation, Gent^R^ | | |  | This study | |  |
| pMQ30*^MRR1-Y1126N^* | DH3833 | pMQ30-*MRR1^Y1126N^-HygB* complementation, Gent^R^ | |  | | This study | |  |
| pMQ30*^MRR1-Y1126N+P1174P(t)^* | DH3834 | pMQ30-*MRR1^Y1126N+P1174P(t)^-HygB* complementation, Gent^R^ | | |  | This study | |  |
| pMQ30*^MRR1-359*+Y1126N^* | DH3835 | pMQ30-*MRR1^S359*+Y1126N^- HygB* complementation, Gent^R^ | |  | | This study | |  |
| pMQ30*^MRR1-P1174P(t)^* | DH3836 | pMQ30-*MRR1^P1174P(t)^- HygB* complementation, Gent^R^ | |  | | This study | |  |
| pMQ30*^MRR1-L1191H^* | DH3837 | pMQ30-*MRR1^L1191H^- HygB* complementation, Gent^R^ | |  | | This study | |  |
| pMQ30*^MRR1-Q1197*^* | DH3838 | pMQ30-*MRR1^Q1197*^- HygB* complementation, Gent^R^ | |  | | This study | |  |
| pNAT | DH2664 | TEF1p*-NAT1*, Amp^R^ | |  | | (12) | |  |
| pYM70 | DH3352 | TEF2p-*HygB*, Amp^R^ | |  | | (13) | |  |
| pNAT-Chr4 neutral | DH3261 | *NAT1* w/ flanks targeting intragenic site on Chr 4, Amp^R^ | |  | | This study | |  |
| pMQ30 | DH2620 | Plasmid that replicates in *S. cerevisiae* and *E. coli*, using uracil or gentamycin selection, respectively. | |  | | (14) | |  |
| pRS426 | DH2958 | Cloning plasmid | |  | | (15) | |  |

*Dartmouth Hitchcock Medical Center, Lebanon, NH (DHMC)

**Supplemental references**

1. Grahl N, Demers EG, Crocker AW, Hogan DA. 2017. Use of RNA-protein complexes for genome editing in non-*albicans Candida* species. mSphere 2.

2. Demers EG, Biermann AR, Masonjones S, Crocker AW, Ashare A, Stajich JE, Hogan DA. 2018. Evolution of drug resistance in an antifungal-naive chronic *Candida lusitaniae* infection. Proc Natl Acad Sci U S A 115:12040-12045.

3. Biermann AR, Demers EG, Hogan DA. 2020. Mrr1 regulation of methylglyoxal catabolism and methylglyoxal-induced fluconazole resistance in *Candida lusitaniae*. Mol Microbiol doi:10.1111/mmi.14604.

4. Butler G, Rasmussen MD, Lin MF, Santos MA, Sakthikumar S, Munro CA, Rheinbay E, Grabherr M, Forche A, Reedy JL, Agrafioti I, Arnaud MB, Bates S, Brown AJ, Brunke S, Costanzo MC, Fitzpatrick DA, de Groot PW, Harris D, Hoyer LL, Hube B, Klis FM, Kodira C, Lennard N, Logue ME, Martin R, Neiman AM, Nikolaou E, Quail MA, Quinn J, Santos MC, Schmitzberger FF, Sherlock G, Shah P, Silverstein KA, Skrzypek MS, Soll D, Staggs R, Stansfield I, Stumpf MP, Sudbery PE, Srikantha T, Zeng Q, Berman J, Berriman M, Heitman J, Gow NA, Lorenz MC, Birren BW, Kellis M, et al. 2009. Evolution of pathogenicity and sexual reproduction in eight *Candida* genomes. Nature 459:657-62.

5. Francois F, Chapeland-Leclerc F, Villard J, Noel T. 2004. Development of an integrative transformation system for the opportunistic pathogenic yeast *Candida lusitaniae* using *URA3* as a selection marker. Yeast 21:95-106.

6. Morschhauser J, Barker KS, Liu TT, Bla BWJ, Homayouni R, Rogers PD. 2007. The transcription factor Mrr1p controls expression of the *MDR1* efflux pump and mediates multidrug resistance in *Candida albicans*. PLoS Pathog 3:e164.

7. Hampe IAI, Friedman J, Edgerton M, Morschhauser J. 2017. An acquired mechanism of antifungal drug resistance simultaneously enables *Candida albicans* to escape from intrinsic host defenses. PLoS Pathog 13:e1006655.

8. Liu Z, Myers LC. 2017. *Candida* *albicans* Swi/Snf and mediator complexes differentially regulate Mrr1-induced *MDR1* expression and fluconazole resistance. Antimicrob Agents Chemother 61.

9. Sullivan DJ, Westerneng TJ, Haynes KA, Bennett DE, Coleman DC. 1995. *Candida dubliniensis* sp. nov.: phenotypic and molecular characterization of a novel species associated with oral candidosis in HIV-infected individuals. Microbiology 141 ( Pt 7):1507-21.

10. Moran GP, Sullivan DJ, Henman MC, McCreary CE, Harrington BJ, Shanley DB, Coleman DC. 1997. Antifungal drug susceptibilities of oral *Candida dubliniensis* isolates from human immunodeficiency virus (HIV)-infected and non-HIV-infected subjects and generation of stable fluconazole-resistant derivatives *in vitro*. Antimicrob Agents Chemother 41:617-23.

11. Moran GP, Sanglard D, Donnelly SM, Shanley DB, Sullivan DJ, Coleman DC. 1998. Identification and expression of multidrug transporters responsible for fluconazole resistance in *Candida dubliniensis*. Antimicrob Agents Chemother 42:1819-30.

12. Min K, Ichikawa Y, Woolford CA, Mitchell AP. 2016. *Candida albicans* gene deletion with a transient CRISPR-Cas9 system. mSphere 1.

13. Basso LR, Jr., Bartiss A, Mao Y, Gast CE, Coelho PS, Snyder M, Wong B. 2010. Transformation of *Candida albicans* with a synthetic hygromycin B resistance gene. Yeast 27:1039-48.

14. Shanks RM, Caiazza NC, Hinsa SM, Toutain CM, O'Toole GA. 2006. *Saccharomyces cerevisiae*-based molecular tool kit for manipulation of genes from gram-negative bacteria. Appl Environ Microbiol 72:5027-36.

15. Christianson TW, Sikorski RS, Dante M, Shero JH, Hieter P. 1992. Multifunctional yeast high-copy-number shuttle vectors. Gene 110:119-22.
