# Supplementary material for: Balancing Positive and Negative Selection: In Vivo Evolution of Candida lusitaniae MRR1
Source: mBio. 2021 Mar 30;12(2):e03328-20. doi: 10.1128/mBio.03328-20 (PMC8092287; doi:10.1128/mBio.03328-20)
Supplement: TABLE S4 [file mBio.03328-20-st004.docx]

| **Table S4: Oligos and primers used in this study.** | | | | |
| --- | --- | --- | --- | --- |
| **Name** | **Description** | **Sequence** | | |
| **Primers for making stitched KO constructs** | | | | |
| AB001 | Left flank of knockout construct for *MRR1* FWD | AAGGCGTGTCCTTCATGTT | | |
| AB003 | Left flank of knockout construct for *MRR1* REV w/ homology to *NAT1* | AACGTCGTGACTGGGAAAAATCATTAGCTTCGCTGGAATTTCTGTTT | | |
| AB004 | Right flank of knockout construct for *MRR1* FWD w/ homology to *NAT1* | TATCCGCTCACAATTCCACTGCTCGGTTCTGGTTCTATATG | | |
| AB006 | Right flank of knockout construct for *MRR1* REV | GAGTACGTGGATCTCTACTTGATG | | |
| AB007 | Nested to amplify across stitched *MRR1* knockout construct FWD | CTTTGCTTGTTTGGGAAACCTC | | |
| AB008 | Nested to amplify across stitched *MRR1* knockout construct REV | TTTCCGGGTTCAATGCCA | | |
| AB009 | Amplify *NAT1* FWD w/ homology to *MRR1* for knockout construct | AAACAGAAATTCCAGCGAAGCTA  ATGATTTTTCCCAGTCACGACGTT | | |
| AB010 | Amplify *NAT1* REV w/ homology to *MRR1* for knockout construct | CATATAGAACCAGAACCGAGCAG  TGGAATTGTGAGCGGATA | | |
| ED038 | Left flank of knockout construct for *MDR1* FWD | CAGTAGTGTGTTCGTCTCCTTAG | | |
| ED039 | Left flank of knockout construct for *MDR1* REV w/ homology to *NAT1* | AACGTCGTGACTGGGAAAAATCATTA GCGATTAGGTATTAGATGGATGTTTG | | |
| ED042 | Nested to amplify across stitched *MDR1* knockout construct FWD | CGGCGGAGTTATATCCGTTTC | | |
| ED043 | Nested to amplify across stitched *MDR1* knockout construct REV | GGCTTCCGTATTTAAGCTGTACT | | |
| ED044 | Amplify *NAT1* FWD w/ homology to *MDR1* for knockout construct | CAAACATCCATCTAATACCTAAT  CGCTAATGATTTTTCCCAGTCACGACGTT | | |
| ED046 | Right flank of knockout construct for *MDR1* FWD w/ homology to *NAT1* | TAT CCG CTC ACA ATT CCA C GAG  TTCACAAGGTAATTGTTCAGG | | |
| ED048 | Right flank of knockout construct for *MDR1* REV | CCGACCCTCCCATTCAATC | | |
| ED049 | Amplify *NAT1* REV w/ homology to *MDR1* for knockout construct | CCTGAACAATTACCTTGTGAACT  CGTGGAATTGTGAGCGGATA | | |
| ED187 | Left flank of knockout construct for *MDR1* REV w/ homology to *HygB* | GCAATATCGAACAGCAAGCACTATAGCG  ATTAGGTATTAGATGGATGTTTG | | |
| ED188 | Amplify *HygB* FWD w/ homology to *MDR1* for knockout construct | CAAACATCCATCTAATACCTAATCGCTATAGTGCTTGCTGTTCGATATTGC | | |
| ED189 | Amplify *HygB* REV w/ homology to *MDR1* for knockout construct | CCTGAACAATTACCTTGTGAACTCATTTTATGATGGAATGAATGGG | | |
| ED190 | Right flank of knockout construct for *MDR1* FWD w/ homology to *HygB* | CCCATTCATTCCATCATAAAATGAGTTCACAAGGTAATTGTTCAGG | | |
| AB069 | Left flank of knockout construct for *CAP1* FWD | TCAACAGAAGTAGTGCCTGTAT | | |
| AB092 | Left flank of knockout construct for *CAP1* REV w/ homology to *HygB* | GACGTCAGGTGGCACTTTTCGGGGGCTTTAACGGCAAGGAGTTAG | | |
| AB095 | Right flank of knockout construct for *CAP1* FWD w/ homology to *HygB* | AATAGGCGTATCACGAGGCCGAAACGGACAGCGTAGTTAGT | | |
| AB072 | Right flank of knockout construct for *CAP1* REV | CAGCTTCTCCGTGTATCGTTTA | | |
| AB073 | Nested to amplify across stitched *CAP1* knockout construct FWD | CGCTTCTTTACGCATTGTAACC | | |
| AB074 | Nested to amplify across stitched *CAP1* knockout construct REV | CAGCGTATTCGACCCATCTT | | |
| AB093 | Amplify *HygB* FWD w/ homology to *CAP1* for knockout construct | CTAACTCCTTGCCGTTAAAGCCCCCGAAAAGTGCCACCTGACGTC | | |
| AB094 | Amplify *HygB* REV w/ homology to *CAP1* for knockout construct | ACTAACTACGCTGTCCGTTTCGGCCTCGTGATACGCCTATT | | |
| **Primers for making *NAT1* knock-in plasmids** | | | | |
| POP42 | Left flank of knock-in construct for *NAT1* at Chr4 neutral site FWD | GACTCACTATAGGGCGAATTGGGGTACCACACGGGGAGAAAGTTTCACAG | | |
| POP43 | Left flank of knock-in construct for *NAT1* at Chr4 neutral site REV | CAGTAACACCATTCGTACTCTAGCGCCTAGCATGCC | | |
| POP44.2 | Amplify *NAT1* FWD w/ homology to Chr4 neutral site | GGTCACCCGGCCAGCGACATGGGGTCACCCGGCCAGCGACATG | | |
| POP45 | Amplify *NAT1* REV w/ homology to Chr4 neutral site | GCTTATTCATTTCCACAGGTCTAGAATACTCAAGCTATGCATCCAACGCG | | |
| POP46 | Right flank of knock-in construct for *NAT1* at Chr4 neutral site FWD | CTAGACCTGTGGAAATGAATAAGC | | |
| POP47 | Right flank of knock-in construct for *NAT1* at Chr4 neutral site REV | ATCGATACCGTCGAGTCGACCCAACTCTTGTATTTTCTCCATCCC | | |
| **Primers for making *MRR1* complementation constructs** | | | | |
| ED103 | Amplify *MRR1* and 1kb upstream w/ homology to pMQ30 FWD | TTTTCCCAGTCACGACGTTGTAAAACGACGGCCGCGGCCGCAAGGCGTGTCCTTCATGTT | | |
| ED110 | Amplify 1 kb downstream *MRR1* w/ homology to pMQ30 REV | CGGATAACAATTTCACACAGGAAACAGCTATGACCCGGAGCTTTTCATCACCACCA | | |
| ED115 | Amplify *MRR1* and 1kb upstream w/ homology to *HygB* REV | AGCAATATCGAACAGCAAGCACTATATCT  AGAGGTTTACGACGGAACTAGCTGCT | | |
| ED121 | Amplify *HygB* w/ homology to *MRR1* FWD | TAGTTCAACTCAGCAGCTAGTTCCGTCGTAAACCTCTAGATATAGTGCTTGCTGTTCGAT | | |
| ED122 | Amplify *HygB* w/ homology to *MRR1* downstream REV | CTGATGTGCCGATCAATGAGTCAGAAACAGCCTGTATTTTATGATGGAATGAATGGGATG | | |
| ED127 | Amplify *MRR1* from amino acids 1064 FWD (remove Y1126N) | CTTGGAGGATTACCAAAGGTCAAACATT  CATGTTGAAGACTG | | |
| ED128 | Amplify *MRR1* from amino acid 1164 REV (remove Y1126N) | TGGCACTGAAGCCACTAGTACTAGTA  GATTCGCTGGAATTGC | | |
| ED129 | Amplify *MRR1* from amino acid 1164 FWD (isolate P1174P) | GCAATTCCAGCGAATCTACTAGTACTAGTGGCTTCAGTGCCA | | |
| POP218 | Amplify L1191H alone FWD | AGGAGGCGAAAATCTCCTTGACAAGGAAGCAGATCGCCTTATAGTTGATCG | | |
| POP219 | Amplify L1191H alone REV | TGAGTTGAACTAAGAAATTGATATTAATTAAgTAATAAAATCATCCATGACATTAGAGTATTTCAATCACTCAG | | |
| POP220 | Amplify Q1197* alone FWD | AGGAGGCGAAAATCTCCTTGACAAGGAAGCAGATCGCCTTATAGTTGATCG | | |
| POP221 | Amplify Q1197* alone REV | TGAGTTGAACTAAGAAATTGATATTAATTAAGTAATAAAATCATCCATGACATTAGAGTATTTCAATCACTCAG | | |
| **Primers for qRT-PCR** | | |  |  |
| ED058 | Forward for RT-PCR of M*DR1* | TCCATCCATGGGTCCATTATTC | | |
| ED059 | Reverse for RT-PCR of M*DR1* | CTCAACACAAGGAAAGCACATC | | |
| ED060 | Forward for RT-PCR of *ACT1* | GGTAGAGACTTGACCGACTACTT | | |
| ED061 | Reverse for RT-PCR of *ACT1* | CCTTGATGTCACGGACGATTT | | |
| AB039 | Forward for RT-PCR of *MGD1* | CGCAGAAATCCCTAAAGTAAA | | |
| AB040 | Reverse for RT-PCR of *MGD1* | TACCCTTTGCTTCGTTCTT | | |
| ED090 | Forward for RT-PCR of *FLU1* | AGTGCTACAACCAGAGGTAATG | | |
| ED091 | Reverse for RT-PCR of *FLU1* | CCGTGAAACCACCACAAATG | | |
| **crRNA for CRISRP-CAS9 mediated mutagenesis** | | | | |
|  | crRNA for *NAT1* | GGGAAAACCTTAGTCAATGG | | |
|  | crRNA for *MDR1 (CLUG_01938/39)* | AGTCCTTGCTTGGCCACAGG | | |
|  | crRNA for *MRR1 (CLUG_00542)* | TTCATCACTAAAGATGATGG | | |
|  | crRNA for *CAP1* (*CLUG_02670*) | AACCACACACAAAACCAGG | | |
|  | crRNA for neutral site on Chr4 | CCAAAGTTCCAAGTCTGTGG | | |
| **Synthesized gene fragments*** | | | | |
| ^a^Partial *MRR1-L1191H* | AAAATCTCCTTGACAAGGAAGCAGATCGCCTTATAGTTGATCGTTTCAACACATGTACATTTTGGTATCCGGACATTCGAAGGATATTGAAACTTACGCTTAATGTCAATGGTAGAGTTCCTTTACCTGAATTATGCTCGTTGTTATCTAATGCCGAGTTAGACTGGTTTCAAAAATGTGGTACTCTTTCAGAAGCCCTCCAATGTGGAGGATTAGGTGTTAAGTCTACTGTCGAAAGAAATTACACCGTCAAGATTTATCTTGCGTTGAGGGTGTCATTCCTCGCCATATTCTTCCACATTTTTCTCCATTATGAACGGAAGAATGACCACGTTTCCTTCTTTTACTTGAAAAAATGTTTGCTCATTACTACTGCTGATATCATGCCTCACTATGAGACTCTCTTGTGCAAGAGTGAAGTTGTCAGTGATATGATTATCAACCCTACTCTTGAAATGGCTGTGCACAAGGCAAACATTATATACCTTGCGGCCATTATTCGTGTTAACTTTGCCGTGTATCACTTGAGACAATCTTCAGAGCACGACCAAAGATGCAAAAATGATAAGCAATACTTGACATACTTCCAAAAATTGTGCCAACTCTCGTCTTGTCTTACTAGAGCAAGTGAGTACAGTATTTCTGTCATTTCCAAAATCAGCAATCGGTACTACTACGCTTGGAGGATTACCAAAGGTCAAACATTCATGTTGAAGACTGTAACGTCGACACAATTCTATGAGTCAAATTACCACGCCGCCTACCTGCTATACTCAACAAGGTTTTCTTGTCAACAGATTGATGAGTTGATCTGCATTTGTGAAACCACTTTAAGCAAGTTCCGCCACACAGAATTCAGAACTTATGGATTTTCGAGGGAAGTGAACGACCAGTTGGTCAAATGTCAGCAGTATTCGTGTGACCCAGTACGCAACGCAAGCAATTCCAGCGAATCTACTAGTACTAGTGGCTTCAGTGCCAGTACTGATTCTATCTCCACCGATGACCCTACTAACCGGGTGACCAATACGGAAGTTGACAAATTGTGGTTGCAGCTCC**A**TTCGATGAAGCATGATCAGCTTTTCAATGAGGATTACCGTGAAGCGCCCGAGGTGATGGTGACAAATGGGAACGGCACAACCAACAAACCCAATAGTCAGAATCACGAGGCGAATGCTGGAGCGGCGACAACAAACGATTTTGCTCGCTTTGGTTACGACATGGAGATGGAGAACAGATATGACTGTTTCAGTGACCTCCCATTTGATCAGGTATTTAATTTCTAGTTATGGAACGAAATTTGGCAAGGATcCTTTACTGGTAAAGGGTATGTTTAAGACATGAAAGCATTTGCAAATTTTTTGTGCTCGGTTCTGGTTCTATATGAGCACATACAAATCTGAGTGATTGAAATACTCTAATGTCATGGATGATTTTATTACTTAATTAATATCAAT | | | |
| ^a^Partial *MRR1-Q1197** | CTCCTTGACAAGGAAGCAGATCGCCTTATAGTTGATCGTTTCAACACATGTACATTTTGGTATCCGGACATTCGAAGGATATTGAAACTTACGCTTAATGTCAATGGTAGAGTTCCTTTACCTGAATTATGCTCGTTGTTATCTAATGCCGAGTTAGACTGGTTTCAAAAATGTGGTACTCTTTCAGAAGCCCTCCAATGTGGAGGATTAGGTGTTAAGTCTACTGTCGAAAGAAATTACACCGTCAAGATTTATCTTGCGTTGAGGGTGTCATTCCTCGCCATATTCTTCCACATTTTTCTCCATTATGAACGGAAGAATGACCACGTTTCCTTCTTTTACTTGAAAAAATGTTTGCTCATTACTACTGCTGATATCATGCCTCACTATGAGACTCTCTTGTGCAAGAGTGAAGTTGTCAGTGATATGATTATCAACCCTACTCTTGAAATGGCTGTGCACAAGGCAAACATTATATACCTTGCGGCCATTATTCGTGTTAACTTTGCCGTGTATCACTTGAGACAATCTTCAGAGCACGACCAAAGATGCAAAAATGATAAGCAATACTTGACATACTTCCAAAAATTGTGCCAACTCTCGTCTTGTCTTACTAGAGCAAGTGAGTACAGTATTTCTGTCATTTCCAAAATCAGCAATCGGTACTACTACGCTTGGAGGATTACCAAAGGTCAAACATTCATGTTGAAGACTGTAACGTCGACACAATTCTATGAGTCAAATTACCACGCCGCCTACCTGCTATACTCAACAAGGTTTTCTTGTCAACAGATTGATGAGTTGATCTGCATTTGTGAAACCACTTTAAGCAAGTTCCGCCACACAGAATTCAGAACTTATGGATTTTCGAGGGAAGTGAACGACCAGTTGGTCAAATGTCAGCAGTATTCGTGTGACCCAGTACGCAACGCAAGCAATTCCAGCGAATCTACTAGTACTAGTGGCTTCAGTGCCAGTACTGATTCTATCTCCACCGATGACCCTACTAACCGGGTGACCAATACGGAAGTTGACAAATTGTGGTTGCAGCTCCTTTCGATGAAGCATGAT**T**AGCTTTTCAATGAGGATTACCGTGAAGCGCCCGAGGTGATGGTGACAAATGGGAACGGCACAACCAACAAACCCAATAGTCAGAATCACGAGGCGAATGCTGGAGCGGCGACAACAAACGATTTTGCTCGCTTTGGTTACGACATGGAGATGGAGAACAGATATGACTGTTTCAGTGACCTCCCATTTGATCAGGTATTTAATTTCTAGTTATGGAACGAAATTTGGCAAGGATcCTTTACTGGTAAAGGGTATGTTTAAGACATGAAAGCATTTGCAAATTTTTTGTGCTCGGTTCTGGTTCTATATGAGCACATACAAATCTGAGTGATTGAAATACTCTAATGTCATGGATGATTTTATTACTTAATTAATATC | | | |

^a^Red nucleotide was mutated to obtain sequence of interest.
